# Coix Seed Oil Exerts an Anti–Triple-Negative Breast Cancer Effect by Disrupting miR-205/S1PR1 Axis

**DOI:** 10.3389/fphar.2020.529962

**Published:** 2020-09-25

**Authors:** Ting Fang, Yi-Xin Jiang, Long Chen, Ling Huang, Xin-Hui Tian, Yu-Dong Zhou, Dale G. Nagle, Dan-Dan Zhang

**Affiliations:** ^1^ Interdisciplinary Integrative Medicine Research, Shanghai University of Traditional Chinese Medicine, Shanghai, China; ^2^ School of Pharmacy, Fujian University of Traditional Chinese Medicine, Fuzhou, China; ^3^ Experiment Center for Science and Technology, Shanghai University of Traditional Chinese Medicine, Shanghai, China; ^4^ Department of Chemistry and Biochemistry, College of Liberal Arts, University of Mississippi, University, Misissippi, MS, United States; ^5^ Department of BioMolecular Sciences and Research Institute of Pharmaceutical Sciences (RIPS), School of Pharmacy, University of Mississippi, University, Mississippi, MS, United States

**Keywords:** Coix Seed Oil, triple-negative breast cancer, sphingomyelin metabolism, miR-205, Sphingosine-1-phosphate receptors 1

## Abstract

Coix Seed Oil (CSO) possesses a wide range of pharmacological activities. Kanglaite Injection, a commercial product of CSO, has been used clinically as an anticancer drug in China for decades. However, its molecular mechanisms on triple-negative breast cancer (TNBC) remains to be elucidated. In this study, the effect of CSO was evaluated on murine TNBC 4T1 cells and the orthotopic tumor-bearing mouse model and underlying mechanisms were explored. CSO suppressed cell proliferation, colony formation *in vitro*, and tumor growth *in vivo*. miR-205-5p was substantially altered in CSO treated tumor tissues compared to the control group by miRNA-sequencing analysis. Sphingomyelin metabolism (SM) decreased in serum in model group compared to the control group, while it increased by CSO administration by lipid metabolomics analysis. The expression of sphingosine 1 phosphate receptor 1 (S1PR1), the critical effector of SM, was downregulated upon CSO treatment. Mechanically, miRNA-205 directly targeted S1PR1 to regulate SM and cell proliferation. CSO reduced the expression of S1PR1, cyclinD1, and phosphorylation levels of STAT3, MAPK, and AKT while upregulated p27. These results revealed that CSO exerted an anti-TNBC effect *via* the miR-205/S1PR1 axis to regulate sphingomyelin metabolism, and the downstream STAT3/MAPK/AKT signal pathways were partly involved.

## Introduction

Breast cancer still incurs high morbidity and mortality globally and poses a severe health threat to women in recent years (Bray et al., 2018; Miller et al., 2019). Since triple-negative breast cancer (TNBC) lacks estrogen receptor (ER), progesterone receptor (PR), and human epidermal growth factor receptor (HER2), therapeutic options for TNBC are limited and leaving poor prognosis. TNBC is the most aggressive type with high clinical metastatic rates to bone, liver, brain, lung, and other organs in the late-stage of patients.

Currently, cytotoxic chemotherapy plays an essential role in treating TNBC patients with severe side effects, drug resistance, and poor patient compliance. Traditional Chinese Medicine (TCM) presents advantages including reducing toxicity and side effects, increasing the sensitivity of drugs, and improving immune responses in numerous cancers treatments.

Coix Seed Oil (CSO) is extracted from Coix seed (also named as Job’s Tears seed or adlay), a kind of typical food and drug in China. Kanglaite Injection^®^ (KLT) (Lu et al., 2008), a micro-emulsified injectable product derived from CSO, has been widely used to treat various tumors such as NSCLC (Duan, 2018; Wu et al., 2018), hepatocellular carcinoma (Yang et al., 2018), breast cancer (Guo et al., 2008), pancreatic (Liu et al., 2014; Schwartzberg et al., 2017) and gastric cancer (Zhang et al., 2017). KLT presented antineoplastic effect, reduced the side effects, and improved the quality of life such as cancer pain relief, cancer cachexia syndrome in patients (Liu et al., 2019; Zhang et al., 2019). Current pharmacological reports revealed KLT could induce apoptosis, block the cell cycle, inhibit angiogenesis, reverse multidrug resistance, and enhance the body’s immune function *via* NF-κB and PI3K/Akt/mTOR signaling pathway (Liu et al., 2014) and regulation of oncomir hsa-miR-21 in serum (Wu et al., 2018). However, its exact antitumor mechanism, especially in TNBC, is still unclear.

In this study, we investigated the tumor-suppressive effect of CSO without excipient on TNBC and its potential mechanisms of action.

## Materials and Methods

### Cell Culture and Reagents

The murine mammary carcinoma cell line 4T1-luc2 expressed luciferase was obtained from PerkinElmer. The human TNBC cell line MDA-MB-231 was obtained from the Type Culture Collection of the Chinese Academy of Sciences (Shanghai, China). Cells were maintained in DMEM medium (Gibco, Grand Island, NY, USA) supplemented with 10% fetal bovine serum (FBS; Gibco, Grand Island, NY, USA) at 37°C in a humidified environment (5% CO_2_ and 95% Air).

CSO (Batch Number:190109) was obtained from Zhejiang Kanglaite Pharmaceutical Co., Ltd. (Zhejiang, China). The 3-[4,5-dimethylthiazol-2-yl]-2,5-diphenyltetrazolium bromide (MTT) and dimethyl sulfoxide (DMSO) were from Sigma-Aldrich (St. Louis, MO, USA). Antibodies against S1PR1 (55133-1-AP) and GAPDH (10494-1-AP) were purchased from Proteintech (Chicago, IL, USA). Antibodies against STAT3 (#12640), p-STAT3 (#9145), AKT (#9272), p-AKT (#13038), ERK1/2 (#9102), p-ERK1/2 (#9101), JNK (#9252), p-JNK (#9251), P38 (#9212), and p-P38 (#9211) were obtained from Cell Signaling Technology (Boston, MA, USA). Antibodies against Cyclin D1 (ab134175) and p27 (ab32034) were obtained from Abcam (Cambridge, MA, USA). Trizol Reagent, RNAIMAX reagent was purchased from Invitrogen (Carlsbad, CA, USA). MicroRNA primers, transcription kit, and universal PCR master mix, luciferase reporter plasmids, siRNA-S1PR1, siRNA-NC(negative control) miR-205-5p mimics, miRNA NC (negative control) were purchased from GenePharma (Shanghai, China). Dual-luciferase reporter assay kit was purchased from Promega (Madison, WI, USA)

### Quality Control of CSO by LC-MS

High-resolution liquid-chromatography-mass spectrometry (LC-MS) was performed on Fisher Orbi-Trap Elite (Thermo, Waltham, MA, USA) to detect and analyze the main compounds in CSO.

### MTT Assay

Exponentially grown 4T1 cells were seeded at 2 × 10^3^ cells per well into 96-well plates in a volume of 100 μl and incubated under 37°C and 5% CO_2_ condition overnight. Different concentrations of CSO (125, 250, 500, and 1,000 μg/ml, respectively) and 0.5% ethanol (as vehicle control) added and the incubation continued for another 72 h. A stock solution of MTT (5 mg/ml) was added at 20 μl per well. After 4 h, the supernatant discarded and the precipitate was solubilized in DMSO (100 μl/well). The absorbance at 490 nM measured by SpectraMax190 (Molecular Devices, CA, USA).

### Colony Formation Assay

4T1 cells were seeded into 6-well plates at the density of 300 cells per well and allow to attach overnight and then cultured with different concentrations of CSO. After 24 h, the medium of different groups was replaced with complete medium. The medium of each well was discarded and washed after 7 days. Cells were fixed, washed, and stained with crystal violet (0.5%). The colonies with over 50 cells were imaged and accounted.

### Orthotopic Tumor Bearing Mouse Model

Female BALB/c mice aged 5 weeks were purchased from the Shanghai Slack Laboratory Animals Co., Ltd and housed at the Shanghai University of Traditional Chinese Medicine animal facility. Murine 4T1 cells(1 × 10^6^) were injected into the upper mammary fat pad of mice. After the implantation of cells 7 days, these tumor-bearing mice were randomly divided into the model group and CSO treatment group. Mice of the model group only received vehicle control (4% egg yolks) with daily gavage. The CSO treatment group was administered 3 ml CSO/kg/d in egg yolks suspension with daily gavage. The volume of tumors was measured daily using a Vernier caliper and calculated using the formula V = (ab^2^)/2(the longest diameter a, the shortest diameter b). These mice were weighed and measured the volume of tumors daily. After 14 days of continuous vehicle control or CSO administration, these mice were sacrificed to gain the blood and tumor of each mouse. Another control group (six mice) administered vehicle control was set for the blood samples.

Female BALB/c nude mice aged 5–6 weeks (weight: 18–22 g) were purchased from the Shanghai Slack Laboratory Animals Co., Ltd and housed at the Shanghai University of Traditional Chinese Medicine animal facility. Human TNBC MDA-MB-231 cells were resuspended at a density of 1x10^8^ cells/ml. Five million cells (50 μl) of mixed Matrigel (50 μl) were injected into the second mammary fat pad of mice. When the tumors volume of these mice reached around 200 mm^3^, the mice were randomly divided into the model group and CSO treatment group. The treatment group was given CSO suspended in egg yolk intragastrically at a dose of 6 ml CSO/kg/d, and the model group was given vehicle control (4% egg yolk). The tumor volume and the bodyweight of these mice were recorded daily. These mice were sacrificed after 21 days of continuous administration.

### Bioluminescent Imaging

D-Luciferin (PerkinElmer, 150 μl in PBS solution) was injected intraperitoneally into mice and imaged under IVIS Imaging System (Xenogen, USA), and Living Image Software (Xenogen) was used to quantify the photons/second(p/s) emitted.

### miRNA Sequence Analysis

miRNA sequence analysis was performed to assess miRNA expression profiles in the paired tumor tissues from the model group and CSO administration group. The fold changes (FCs) in the expression of individual miRNA were calculated. Differentially expressed miRNAs with log2|FC| > 2.0 and P < 0.05 were considered to be significant.

### Lipid Metabolomics Analysis

Blood samples collected from the mice were stored at 4°C for 1 h, centrifuged at 4,000 rpm (4°C, 15 min) by the centrifuge (Eppendorf, Hamburg, Germany), and the supernatant transferred into 1.5-ml centrifuge tubes. Serum sample (50 μl) was diluted with ultrapure Millipore water (1:1 ratio), mixed with chloroform/methanol (2:1 ratio; 200 μl), vortexed, and centrifuged at 12,000 rpm for 10 min at 4°C. The lower organic phase was taken and repeat the operation once. Combine the two organic phases and dry them using vacuum centrifugation. Methanol/isopropanol (1:1 ratio; 200 μl) mixture was added to re-dissolve them, and centrifuge at 1,200 rpm (4°C,10 min). The centrifuged sample (150 μl) was taken to place in the inner tube of the liquid *via* l for the lipidomic test.

Chromatographic conditions: the instrument was HPLC-LTQ-Orbitrap Elite (Thermo, Waltham, MA, USA); column was Thermo Hypersil Gold C18; mobile phase: Phase A (60% water and 40% acetonitrile with 0.1% formic acid, 10 mmol ammonium formate) and Phase B (10% acetonitrile and 90% isopropanol, containing 0.1% formic acid, 10 mmol ammonium formate); elution procedure: 0~1.0 min, 70% A; 1.0~2.0 min, 70%~55% A; 2.0~12.0 min, 55%~35% A; 12.0~18.0 min, 35%~15% A; 18.0~20.0 min, 15%~0% A; 20.0~25.0 min, 100% B; 25.1~30.0 min, 100% B; volume flow was 0.3 ml/min, injection volume was 4 μl; and the column temperature was 40°C.

Mass spectrometry conditions: ion source spray voltages were 3.5 and 3.2 kV in the ESI^+^ and ESI^-^ modes, respectively. The temperature of capillary was set at 350°C with the sheath gas flow rate at 35 arb, aux gas flow at 15 arb, and sweep gas flow rate at 1 arb. The heater temperature was at 300°C. The S-Lens RF level was set at 60. The MS was operated at a resolving power of 6,000 in Full-scan mode (scan range: 100–1,000 *m/z*) and of 15,000 in the top 10 data-dependent MS mode (collision energy: 35) with dynamic exclusion setting of 10 s.

### Immunohistochemistry (IHC)

IHC assay was performed to assess the levels of Ki67 and S1PR1 proteins in tumor samples. The experimental procedure was minor modified as previously reported (You et al., 2018).

### Western Blotting Analysis

Cells or tumor tissues were lysed by RIPA buffer containing phosphatase inhibitors and protease inhibitors. The concentration of protein of each group was measured by BCA assay. Proteins were separated by SDS-PAGE and transferred to the PVDF membrane. Membranes were incubated with special primary or secondary antibodies. After washing with TBST, the ECL kit was used for band detection.

### Transfection

Transfection of miR-205-5p mimic and miRNA-NC into 4T1 cells was performed by RNAIMAX reagent according to the manufacturer’s instructions.

### qRT-PCR Analysis

The total RNA of tumor tissues and cells extracted using the Trizol reagent according to the manufacturer’s instructions. miR-205-5p, miR-200a-3p, miR-429-3p, miR-200c-3p, miR-200b-3p, and miR-141-3p were selected to validate the results of miRNA sequencing analysis.

### Dual-Luciferase Reporter Assay

4T1 cells were co-transfected with miR-205 mimic and a luciferase reporter plasmid containing wild-type S1PR1 3’-UTR or the mutant S1PR1 3’-UTR, respectively. Following 48 h of transfection, luciferase activity was determined by the dual-luciferase reporter assay system (Promega, Madison, WI, USA). Luciferase activity of renilla was normalized to that of firefly luciferase.

### Statistical Analysis

All data were presented as mean ± SD (standard deviation, SD) from three independent experiments. One-way analysis of variance was used among multiple groups of data by SPSS 20.0 statistical software. *P* < 0.05 was considered statistically significant.

## Results

### Main Compounds Identified in CSO by LC-MS

LC-MS was performed to determine the main ingredients in CSO. The results showed six components including 1, 2-Dilinoleoyl-3-palmitoyl-rac-glycerol, 1, 2- Dilinoleoyl-3-oleoyl-rac-glycerol, 1-palmitoyl-2-oleoyl-3-linoleoyl-rac-glycerol, 1, 2-Dioleoyl-3-linoleoyl-rac-glycerol, 1, 2-Dioleoyl-3-palmitoyl-rac-glycerol and Glycerol trioleate existed in CSO. Identified compounds were listed in **Table 1**, and the results of MTT assay were presented in the supplementary materials.

**Table 1:** Measurement results of Coix Seed oil by LC-MS.

NO.	t_R_/min	Compounds	Molecular formula	Molecular mass
1	17.09	1,2-Dilinoleoyl-3-palmitoyl-rac-glycerol	C55H98O6	854
2	17.27	1,2-Dilinoleoyl-3-oleoyl-rac-glycerol	C57H100O6	880
3	17.71	1-palmitoyl-2-oleoyl-3-linoleoyl-rac-glycerol	C55H100O6	856
4	17.85	1,2-Dioleoyl-3-linoleoyl-rac-glycerol	C57H102O6	882
5	18.26	1,2-Dioleoyl-3-palmitoyl-rac-glycerol	C55H102O6	858
6	18.40	Glycerol trioleate	C57H104O6	884

### CSO Inhibited Cell Viability, Colony Formation, and Tumor Growth of 4T1

**Figure 1:**
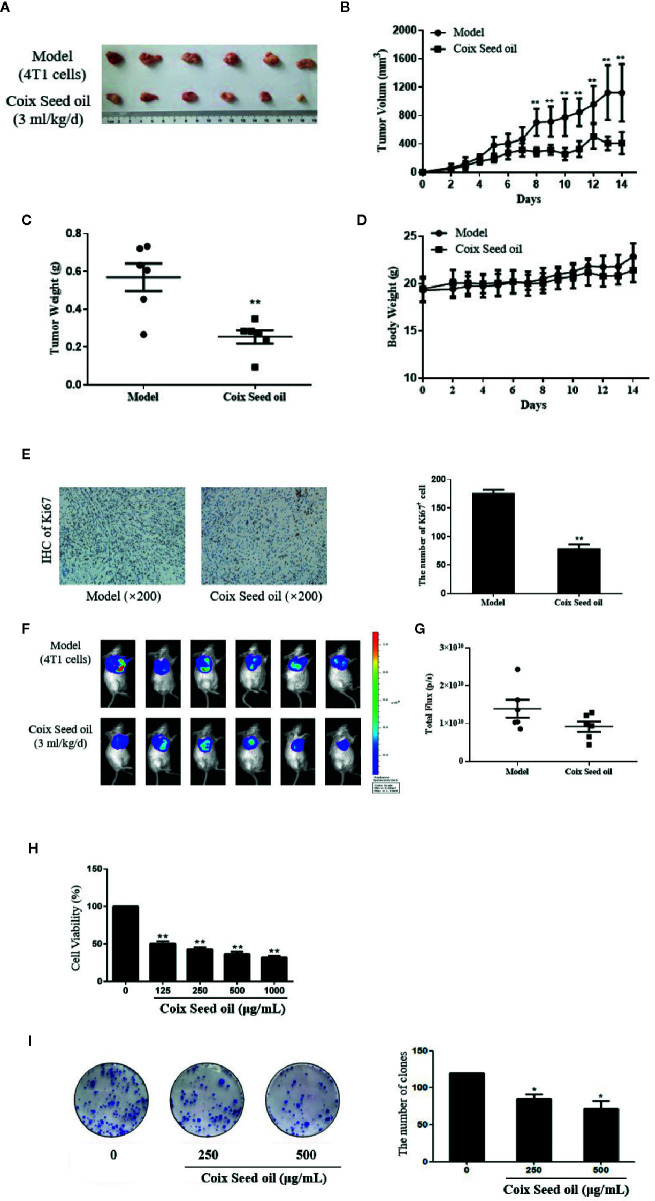
Effect of CSO on tumor growth *in vivo*, and *in vitro* cell viability and colony formation. **(A)** Tumor image. Tumor-bearing BALB/c mice were treated with or without CSO for 14 days. These tumors were excised and imaged. **(B)** Tumor volume. **(C)** Tumor weight. **(D)** Body weight. **(E)** IHC analysis of Ki67. **(F)** Bioluminescence images. **(G)** Total Flux (p/s) of bioluminescence intensity. **(H)** Concentration-dependent effects of CSO on cell viability. **(I)** Concentration-dependent effects of CSO on colony formation. *p < 0.05, **p < 0.01 vs. model.

In order to evaluate the effect of CSO on tumor growth, a triple-negative 4T1 cell-based murine orthotopic transplantation model was used. Compared to the model group, CSO significantly inhibited tumor growth *in vivo*. There was no significant difference in the bodyweight of mice between model and treatment groups, indicating that CSO did no observable toxicity (**Figures 1A–D**). In addition, IHC analysis showed that CSO significantly reduced the number of Ki67-positive cells in tumor tissues (**Figure 1E**). Before the termination of this experiment, we used the IVIS Imaging System to detect the bioluminescence signal of the tumor in these two groups of mice. The luminescence signal of the tumors in the CSO administration group was weaker than that of the model group (**Figures 1F, G**). A nude mouse model based on the triple-negative MDA-MB-231 cell was also used and detailed data has been supplemented in **Figure S3**. These results suggested that CSO can effectively inhibit mammary carcinoma-based tumor growth *in vivo*.

To further investigate the anticancer potential of CSO *in vitro*, we tested CSO on the viability of 4T1 cells and colony formation. The results showed CSO decreased 4T1 cell viability and inhibited 4T1 cell colony formation in a concentration-dependent manner compared to the untreated cells (**Figures 1H, I**).

These results suggested CSO effectively inhibited 4T1 proliferation, colony formation *in vitro*, and tumor growth *in vivo*.

### CSO Strongly Elevated miR-205-5p in Tumor Tissues

**Figure 2:**
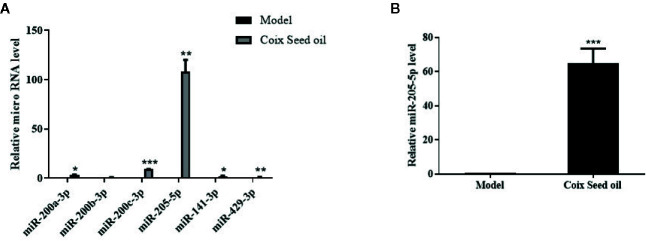
Effect of CSO on the level of miR-205-5p in the tumor tissues of the 4T1 mammary carcinoma allograft mice. **(A)** qRT-PCR analysis was used to detect the expression of miRNAs. Compared with the model group, the levels of miR-200a-3p, miR-200b-3p, miR-200c-3p, miR-205-5p, miR-141-3p, and miR-429-3p increased in the CSO treated group (3 ml/kg, 14 days). **(B)** The expression of miR-205-5p was detected from mixed tumor tissues in two groups by qRT-PCR. Compared with the model group, the level of miR-205-5p from the mixture of tumor tissues increased in the individuals CSO treated (3 ml/kg, 14 days). *P < 0.05, **P < 0.01, ***P < 0.001 vs. model.

miRNA sequencing analysis was carried out to assess the alteration of miRNAs in tumor tissues after CSO administration. The result revealed the top 10 up-regulated miRNA included miR-205-5p, miR-200a-3p, miR-429-3p, miR-200c-3p, miR-200b-3p, miR-203-3p, miR-133b-3p, miR-141-3p, miR-206-3p, and miR-133a-3p and the only down-regulated miR-669f-3p (**Table 2**). Further qRT-PCR analysis of tumor tissue samples confirmed the sequencing results. Among the identified microRNAs, miR-205-5p was most upregulated under CSO treatment among these key miRNAs (**Figures 2A, B**). These results indicated that CSO could strongly increase the level of miR-205-5p in tumor tissues.

**Table 2:** Micro RNA sequencing results.

Micro RNA	Fold Change (Log2)
mmu-miR-205-5p	7.54^**^
mmu-miR-200a-3p	4.48^**^
mmu-miR-429-3p	4.25^**^
mmu-miR-200c-3p	4.01^**^
mmu-miR-200b-3p	3.72^**^
mmu-miR-203-3p	3.57^**^
mmu-miR-133b-3p	3.46^**^
mmu-miR-141-3p	3.35^**^
mmu-miR-206-3p	2.98^**^
mmu-miR-133a-3p	2.52^**^
mmu-miR-669f-3p	−1.55^**^

**P < 0.01 vs. model.

### CSO Increased Sphingomyelin Metabolism (SM) in the Serum

**Figure 3:**
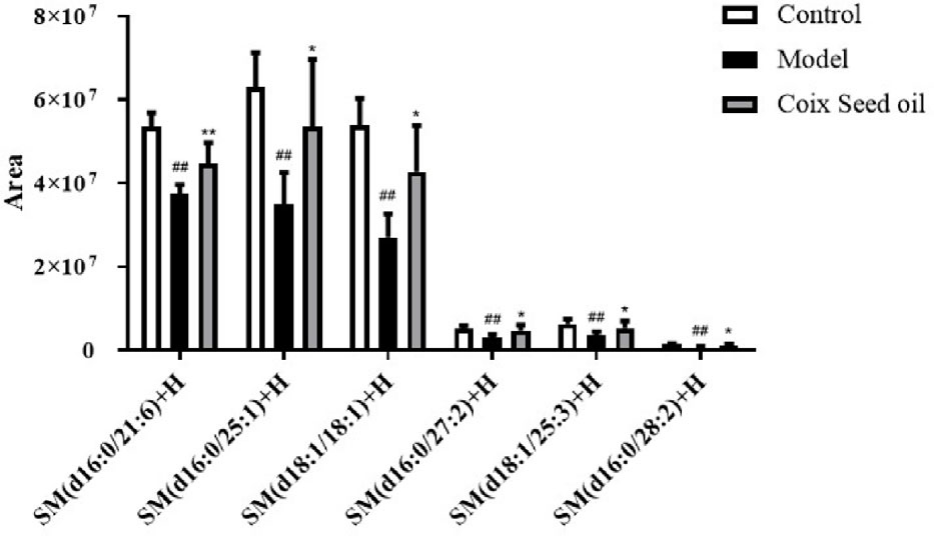
Effect of CSO on the levels of serum sphingomyelin in the 4T1 mammary carcinoma allograft mice. Compared with the control group, the content of sphingomyelin in the serum of the 4T1 allograft mice changed. The sphingomyelin increased in the CSO treatment group(3 ml/kg for 14 days). *^##^p <* 0.01 vs. control and *p < 0.05, **p < 0.01 vs. model.

Due to the oily nature of CSO, we speculated that the tumor-suppressive effect of CSO might be related to lipid metabolism. To test this hypothesis, we performed lipid metabolomics assay and found that compared with normal mouse serum, the level of sphingomyelin in the serum of the 4T1 mammary carcinoma allograft mice decreased significantly, and this phenomenon had a significant callback trend after CSO administration (**Figure 3**). This result demonstrated that the tumor-suppressive effect of CSO was related to the increase of SM.

### miR-205-5p Regulated SM Partly *via* Targeting S1PR1 to Suppress Cell Proliferation

Since miR-205-5p might act as a tumor suppressor miRNA in the development of BC, we transfected miR-205 mimic or miRNA NC into 4T1 cells to compare cell proliferation between these groups.

**Figure 4:**
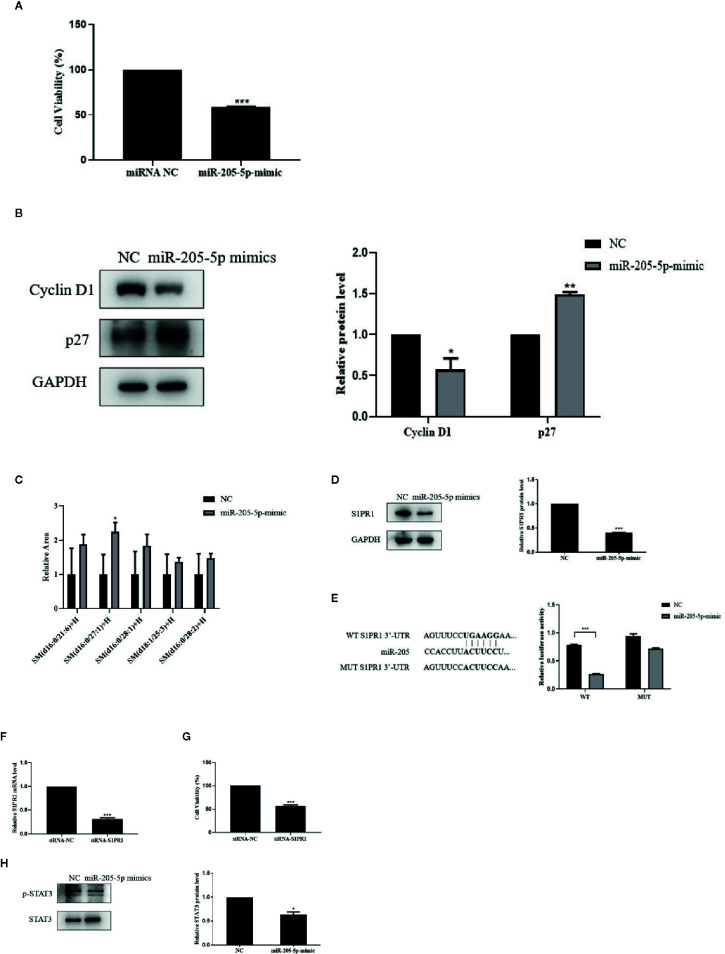
miR-205-5p regulated SM partly *via* targeting S1PR1 to suppress cell proliferation. **(A)** MTT assay of cell viability after transfection of miR-205-5p mimic. **(B)** Protein expressions of cyclinD1 and p27. **(C)** Sphingomyelin levels. **(D)** Expression of S1PR1 protein. **(E)** The potential binding sites of miR-205 in the WT and MUT 3’-UTR of S1PR1 and luciferase reporter assay. **(F)** The gene expression of S1PR1 in siRNA-S1PR1 treated cells. **(G)** MTT assay of cell viability after treated siRNA-S1PR1. **(H)** STAT3 signal pathway. *p < 0.05, **p < 0.01, ***p < 0.001 vs. negative control.

MTT assay result showed that over-expressed miR-205 decreased the ability of cell growth. The expressions of CyclinD1 and p27, the critical mediators in the cell cycle, were regulated by miR-205 after 72-h transfection. These results showed miR-205 could block the cell cycle and proliferation (**Figures 4A, B**).

In order to investigate the correlation between miR-205-5p and SM, we performed the lipidomic analysis on miR-205-5p overexpressed 4T1 cells. Overexpressed miR-205-5p in 4T1 partially increased SM compared to the NC group. Here, we only listed a part of the SM with a higher-fold increase, and these results indicated that miR-205-5p could increase SM accumulation (**Figure 4C**).

We performed bioinformatics analysis to predict targets of miR-205 and found a potential target S1PR1 related to SM in Target RNA Mouse 7.1 (Lewis et al., 2005). Therefore, we examined the protein levels of S1PR1 after miR-205-5p overexpression by western blotting. The up-regulation of miR-205-5p led to the decreased protein expression of S1PR1 compared to the NC group (**Figure 4D**). This result suggested that miR-205-5p could negatively regulate the expression of S1PR1.

In order to validate whether S1PR1 was a target of miR-205, a luciferase reporter assay was carried out. Luciferase activity was reduced in 4T1 cells co-transfected with the wild-type 3’UTR of S1PR1 and miR-205 mimic. However, luciferase activity remained unchanged in the 4T1 cells co-transfected with mutant S1PR1 3’UTR and miR-205 mimic. These data indicated that S1PR1 might be a novel target of miR-205 (**Figure 4E**).

Furthermore, to evaluate the roles of S1PR1 on cell proliferation, we performed MTT assay. After transfection for 72 h, the gene expression of S1PR1 was decreased in siRNA-S1PR1 treated cells compared to the siRNA-NC group (**Figure 4F**). And knockdown of S1PR1 significantly inhibited 4T1 cell proliferation (**Figure 4G**).

S1PR1 exerts its functions *via* downstream signal pathways including STAT3. In addition, STAT3 phosphorylation in 4T1 cells was significantly reduced after miR-205-5p overexpression (**Figure 4H**). This result showed the STAT3 signaling pathway was involved in miR-205-S1PR1-led effects on cell proliferation.

### CSO Reduced S1PR1, CyclinD1 Expressions, and Increased p27 Expression

**Figure 5:**
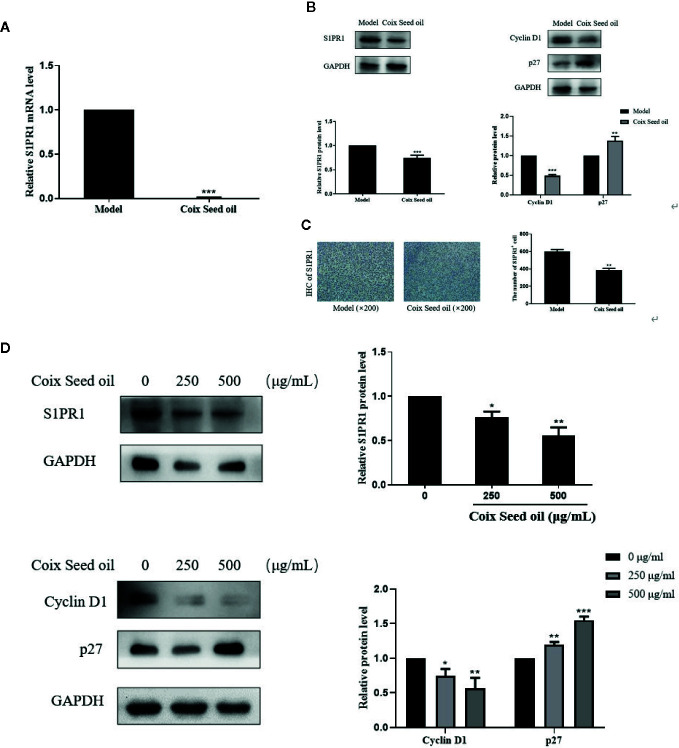
Effects of CSO on S1PR1, Cyclin D1, and p27 expressions in tumor tissues and in cultured cells. **(A)** The gene expression of the S1PR1 in tumor tissues. **(B)** The protein expressions of S1PR1, Cyclin D1, and p27 in tumor tissues. **(C)** IHC analysis of S1PR1 of tumor sections. **(D)** The protein expressions of S1PR1, Cyclin D1, and p27 in 4T1 cells. *p < 0.05, **p < 0.01 ***p < 0.001 vs. untreated.

In order to determine whether CSO can regulate S1PR1, CyclinD1, and p27 expression due to the elevated miR-205, Western blot, qPCR, and IHC were performed to detect the changes of S1PR1 upon CSO treatment. CSO significantly down-regulated S1PR1 and Cyclin D1 expression and upregulated the expression level of p27 in tumor tissues and cells (**Figures 5A, B, D**). Besides, IHC results showed that CSO significantly reduced the expression level of S1PR1 in tumor tissue sections (**Figure 5C**). These results suggested that CSO regulated cell cycle and inhibited tumor growth through the miR-205/S1PR1 axis.

### Proliferation-Related Pathways Were Involved in CSO-Mediated Effects

**Figure 6:**
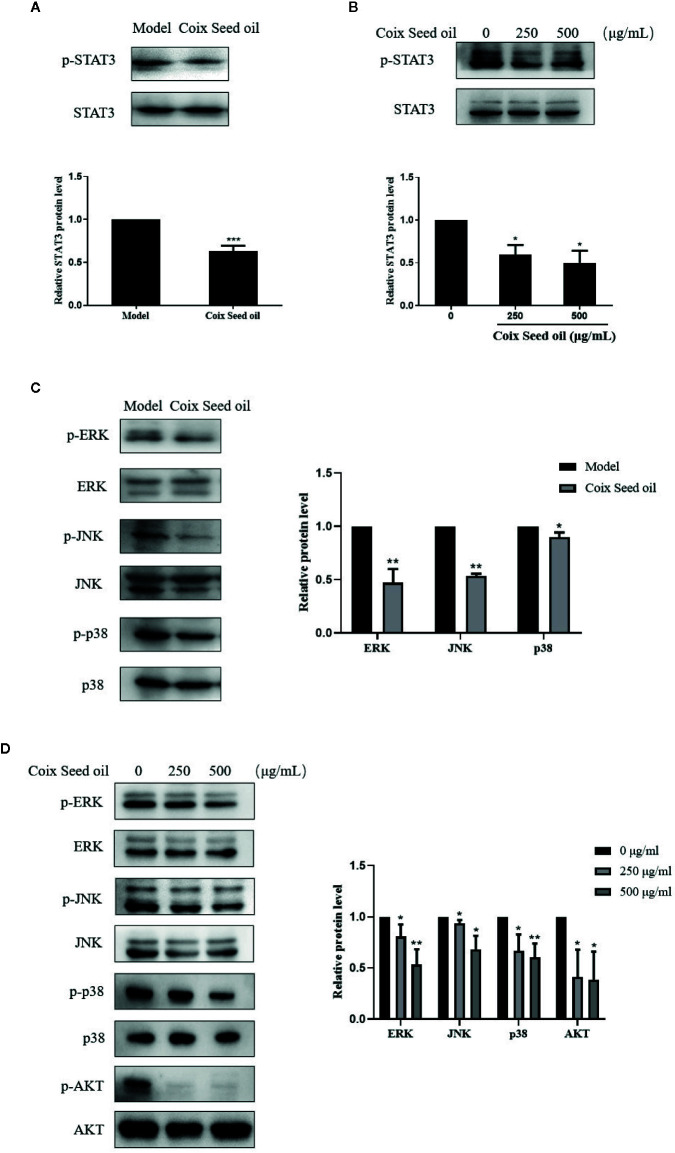
Effects of CSO on proliferation-related pathways in tumor tissues and cells. **(A)** The level of STAT3 phosphorylation in tumor tissues. **(B)** The level of STAT3 phosphorylation in 4T1 cells. **(C)** Phosphorylation levels of ERK, JNK, p38, AKT in tumor tissue. **(D)** Phosphorylation levels of ERK, JNK, p38, and AKT in 4T1 cells. *p < 0.05, **p < 0.01 ***p < 0.001 vs. untreated.

Accumulating studies have confirmed that STAT3 is a transcription regulator of S1PR1 gene expression. Therefore, we tested whether CSO can inhibit the phosphorylation of STAT3 in TNBC by western blotting. Treatment of CSO significantly down-regulated phosphorylation of STAT3 *in vivo* and *in vitro* (**Figures 6A, B**). These data showed that the inactivation of the STAT3 pathway contributed to the downregulation of S1PR1 of the CSO-led effect. We also evaluated its effect on the activation of other proliferation-related pathways such as MAPK and AKT. The treatment with CSO significantly inhibited the phosphorylation of ERK, JNK, p38, and AKT without no effect on the total of ERK, JNK, p38, and AKT *in vivo* and *in vitro* (**Figures 6C, D**). Our results suggest that three important proliferation-related pathways including STAT3, MAPK, and AKT were involved in CSO-led effects.

## Discussion

CSO processes antitumor activities, especially on lung and liver cancers, according to the increasing experimental and clinical evidence. However, the effect of CSO on TNBC and underlying mechanisms related to miRNA alteration remains to be explored. MicroRNA (miRNA) is a type of cellular non-coding small single-stranded RNA that can bind to the complementary sequence on the 3’UTR of specific mRNA, which results in the degradation or unstable condition of the target gene (Croce and Calin, 2005; Kian et al., 2018). In breast cancer, altered expression of specific miRNA may promote or suppress oncogenesis. Among these miRNAs, miR-205-5p abnormally expressed in a variety of tumors. Previous studies showed that miR-205-5p could function as a tumor suppressor in breast cancer (Zhang and Fan, 2015; Wang et al., 2019), prostate cancer (Tucci et al., 2012; Li and Li, 2018), kidney cancer (Chen et al., 2014), and melanoma (Chen et al., 2019), while act as an oncogene in the development of lung cancer (Jiang et al., 2017) and ovarian cancer (Wei et al., 2017). A large amount of evidence proved that the increased expression of miR-205-5p in tumor cells could significantly inhibit the proliferation and metastasis of breast cancer cells, especially TNBC cells (Xiao et al., 2019). Our data showed CSO blocked cell proliferation and tumor growth of TNBC 4T1 cells with an increase of miR-205 by miRNA-sequencing analysis (**Figures 1**, **2**).

The main ingredients in CSO are fatty acids such as palmitic, stearic, oleic, and linoleic acids (Numata et al., 1994), which contribute to its anticancer activity. But the link between CSO and lipid metabolomics in TNBC is still unclear. Recent studies have demonstrated that Altered and reprogrammed sphingomyelin metabolism is strictly related to the progression of numerous cancers and has been identified as a hallmark of cancer aggressiveness. Sphingomyelin and its metabolites take participate in cellular signaling processes including cell growth, differentiation, senescence, and programmed cell death (Ogretmen and Hannun, 2004). Accumulation of sphingomyelins was significantly linked with better prognosis and disease-free survival outcomes in TNBC patients (Purwaha et al., 2018). The result of lipid metabolomics analysis showed CSO administration caused the SM accumulation in tumor-bearing mice (**Figure 3**).

miR-205 is a well-established miRNA in breast cancer. Nevertheless, its function in SM regulation has no previously been reported. Sphingosine-1-phosphate (S1P) and its receptors (sphingosine 1 phosphate receptors, S1PRs) in sphingomyelin metabolism are important signaling effectors involved in regulating cell growth, differentiation, senescence, and death (Pyne and Pyne, 2011; Adada et al., 2016; Ogretmen, 2018). The function of S1P depends on its receptors named S1PRs. Among S1P receptors, S1PR1 is implicated in tumorigenesis and progression (Waters et al., 2003; Brocklyn, 2010; Nagahashi et al., 2014; Weichand et al., 2017; Ogretmen, 2018; Rostami et al., 2019). S1PR1 increased its expression in TNBC. FTY720 reduces tumor growth, metastasis, angiogenesis, and enhances drug-sensitivity of TNBC as an antagonist of S1PR1 (Nagahashi et al., 2018). FTY720 targets the SphK1/S1P/S1PR1 axis including induction of polyubiquitylation and degradation of S1PR1 to regulate sphingolipid metabolism (Graler and Goetzl, 2004). Therefore, S1PR1 emerged as a potential therapeutic target for TNBC.

Our findings confirmed the association between miR-205 and S1PR1 in TNBC cells that S1PR1 is a direct target of miR-205. miR-205 directly targeted S1PR1 to regulate SM and consequent cell proliferation (**Figure 4**) and tumor development.

Cyclin D1 is a tumor-associated protooncogene recognized in recent years and overexpression in various tumor cells (Musgrove et al., 2011). The p27 protein is a tumor suppressor gene discovered in recent years. It blocks the cell transition from the quiescent phase to the proliferative phase in the cell cycle, thereby inhibiting cell proliferation and is a negative regulatory factor of the cell cycle (Chu et al., 2008).

CSO treatment repressed the expression of S1PR1 in cells and tumor tissues accompanied by the downregulated expression of cyclinD1 and upregulated expression of p27 (**Figure 5**).

The S1PR1 signaling can regulate the survival of breast cancer cells, which can increase the survival of breast cancer cells by downregulating the pro-apoptotic protein Bim and upregulating the anti-apoptotic protein Mcl-1, respectively, in ERK and PKC-dependent manner (Rutherford et al., 2013). In addition, one of the main functions of the activation of the S1PR1 signaling pathway is the continuous activation of STAT3 in the form of phosphorylation, leading to overexpression of NF-κB and IL-6, which promotes tumor development (Liang et al., 2013). Studies have shown that STAT3 is a transcription factor for the S1PR1 gene, and enhanced expression of S1PR1 can continuously activate STAT3 and upregulate the expression of the IL-6 gene, thereby accelerating tumor growth and metastasis (Liu et al., 2012; Liang et al., 2013). Similarly, studies indicate that S1PR1 can interact with JAK2, resulting in increased STAT3 phosphorylation, while activated STAT3 can, in turn, upregulate the expression of S1PR1 transcript levels, thus forming a feedforward cycle (Jin et al., 2016). Thus, targeted inhibition of the S1PR1/STAT3 can inhibit tumor proliferation and growth in a STAT3-dependent manner. Experimental evidence implicated S1PR1 coupled to Gi and persistently triggered STAT3, mitogen-activated protein kinase (MAPK) and AKT signaling pathways, to promote many types of tumor growth and metastasis including breast cancer (Lee et al., 2010; Deng et al., 2012; Paik et al., 2014; Go et al., 2015).

Our finding showed miR205-S1PR1 axis regulated activation of the STAT3 signal pathway (**Figures 6A, B**) as well as MAPK and AKT pathways (**Figures 6C, D**).

## Conclusion

CSO exerted an anti-TNBC effect *via* the miR-205/S1PR1 axis to regulate sphingomyelin metabolism, and the downstream STAT3/MAPK/AKT signal pathways were partly involved. CSO may serve as a potential drug for the treatment of TNBC.

## Data Availability Statement

All datasets generated for this study are included in the article/**Supplementary Material**.

## Ethics Statement

The protocol in the animal experiment of this study was reviewed and approved by the Institutional Animal Care and Use Committee of Shanghai University of Traditional Chinese Medicine.

## Author Contributions

TF performed the cell and animal experiments and wrote the manuscript. Y-XJ established the animal model and following CSO administration, and LC performed lipid metabolomics analysis. LH analyzed the data, X-HT performed LC-MS analysis of CSO. Y-DZ and DN modified the manuscript. D-DZ designed this study. All authors contributed to the article and approved the submitted version.

## Funding

This work was supported by the National Natural Science Foundation of China (81773946, 81573673, and 81001666); Innovation project for undergraduates of shanghai university of Traditional Chinese Medicine (2019SHUTCM168).

## Conflict of Interest

The authors declare that the research was conducted in the absence of any commercial or financial relationships that could be construed as a potential conflict of interest.

## References

[B1] Adada M. Luberto C. Canals D. (2016). Inhibitors of the sphingomyelin cycle: Sphingomyelin synthases and sphingomyelinases. Chem. Phys. Lipids 197, 45–59. 10.1016/j.chemphyslip.2015.07.008 26200918

[B2] Bray F. Ferlay J. Soerjomataram I. Siegel R. L. Torre L. A. Jemal A. (2018). Global cancer statistics 2018: GLOBOCAN estimates of incidence and mortality worldwide for 36 cancers in 185 countries. CA Cancer J. Clin. 68 (6), 394–424. 10.3322/caac.21492 30207593

[B3] Brocklyn J. R. (2010). Regulation of cancer cell migration and invasion by sphingosine-1-phosphate. World J. Biol. Chem. 1 (10), 307–312. 10.4331/wjbc.v1.i10.307 21537464 PMC3083934

[B4] Chen Z. Tang Z. Y. He Y. Liu L. F. Li D. J. Chen X. (2014). miRNA-205 is a candidate tumor suppressor that targets ZEB2 in renal cell carcinoma. Oncol. Res. Treat 37 (11), 658–664. 10.1159/000368792 25427583

[B5] Chen Y. Cao K. Li J. Wang A. Sun L. Tang J. (2019). Overexpression of long non-coding RNA NORAD promotes invasion and migration in malignant melanoma via regulating the MIR-205-EGLN2 pathway. Cancer Med. 8 (4), 1744–1754. 10.1002/cam4.2046 30843652 PMC6488211

[B6] Chu I. M. Hengst L. Slingerland J. M. (2008). The Cdk inhibitor p27 in human cancer: prognostic potential and relevance to anticancer therapy. Nat. Rev. Cancer 8 (4), 253–267. 10.1038/nrc2347 18354415

[B7] Croce C. M. Calin G. A. (2005). miRNAs, cancer, and stem cell division. Cell 122 (1), 6–7. 10.1016/j.cell.2005.06.036 16009126

[B8] Deng J. Liu Y. Lee H. Herrmann A. Zhang W. Zhang C. (2012). S1PR1-STAT3 signaling is crucial for myeloid cell colonization at future metastatic sites. Cancer Cell 21 (5), 642–654. 10.1016/j.ccr.2012.03.039 22624714 PMC3360884

[B9] Duan G. C. (2018). The Effects of Combination of Coix Seed Extract and Cisplatin on TAM and Expression of HIF-1alpha in Vivo in Lewis Lung Carcinoma. Iran J. Public Health 47 (6), 838–843. 30087869 PMC6077622

[B10] Go H. Kim P. J. Jeon Y. K. Cho Y. M. Kim K. Park B. H. (2015). Sphingosine-1-phosphate receptor 1 (S1PR1) expression in non-muscle invasive urothelial carcinoma: Association with poor clinical outcome and potential therapeutic target. Eur. J. Cancer 51 (14), 1937–1945. 10.1016/j.ejca.2015.07.021 26238015

[B11] Graler M. H. Goetzl E. J. (2004). The immunosuppressant FTY720 down-regulates sphingosine 1-phosphate G-protein-coupled receptors. FASEB J. 18 (3), 551–553. 10.1096/fj.03-0910fje 14715694

[B12] Guo H. Y. Cai Y. Yang X. M. Wang Z. H. Wang J. L. Zhao X. M. (2008). Randomized phase II trial on mitomycin-C/cisplatin +/- KLT in heavily pretreated advanced breast cancer. Am. J. Chin. Med. 36 (4), 665–674. 10.1142/S0192415X08006132 18711764

[B13] Jiang M. Zhong T. Zhang W. Xiao Z. Hu G. Zhou H. (2017). Reduced expression of miR2055p promotes apoptosis and inhibits proliferation and invasion in lung cancer A549 cells by upregulation of ZEB2 and downregulation of erbB3. Mol. Med. Rep. 15 (5), 3231–3238. 10.3892/mmr.2017.6398 28350117

[B14] Jin L. Liu W. R. Tian M. X. Fan J. Shi Y. H. (2016). The SphKs/S1P/S1PR1 axis in immunity and cancer: more ore to be mined. World J. Surg. Oncol. 14, 131. 10.1186/s12957-016-0884-7 PMC4850705 27129720

[B15] Kian R. Moradi S. Ghorbian S. (2018). Role of components of microRNA machinery in carcinogenesis. Exp. Oncol. 40 (1), 2–9. 10.31768/2312-8852.2018.40(1):2-9 29600985

[B16] Lee H. Deng J. Kujawski M. Yang C. Liu Y. Herrmann A. (2010). STAT3-induced S1PR1 expression is crucial for persistent STAT3 activation in tumors. Nat. Med. 16 (12), 1421–1428. 10.1038/nm.2250 21102457 PMC3088498

[B17] Lewis B. P. Burge C. B. Bartel D. P. (2005). Conserved seed pairing, often flanked by adenosines, indicates that thousands of human genes are microRNA targets. Cell 120 (1), 15–20. 10.1016/j.cell.2004.12.035 15652477

[B18] Li L. Li S. (2018). miR-205-5p inhibits cell migration and invasion in prostatic carcinoma by targeting ZEB1. Oncol. Lett. 16 (2), 1715–1721. 10.3892/ol.2018.8862 30008858 PMC6036508

[B19] Liang J. Nagahashi M. Kim E. Y. Harikumar K. B. Yamada A. Huang W. C. (2013). Sphingosine-1-phosphate links persistent STAT3 activation, chronic intestinal inflammation, and development of colitis-associated cancer. Cancer Cell 23 (1), 107–120. 10.1016/j.ccr.2012.11.013 23273921 PMC3578577

[B20] Liu Y. Deng J. Wang L. Lee H. Armstrong B. Scuto A. (2012). S1PR1 is an effective target to block STAT3 signaling in activated B cell-like diffuse large B-cell lymphoma. Blood 120 (7), 1458–1465. 10.1182/blood-2011-12-399030 22745305 PMC3423784

[B21] Liu Y. Zhang W. Wang X. J. Liu S. (2014). Antitumor effect of Kanglaite(R) injection in human pancreatic cancer xenografts. BMC Complement. Altern. Med. 14, 228. 10.1186/1472-6882-14-228 25005526 PMC4105135

[B22] Liu H. Li L. Zou J. Zhou T. Wang B. Sun H. (2019). Coix seed oil ameliorates cancer cachexia by counteracting muscle loss and fat lipolysis. BMC Complement. Altern. Med. 19 (1), 267. 10.1186/s12906-019-2684-4 31615487 PMC6792186

[B23] Lu Y. Li C. S. Dong Q. (2008). Chinese herb related molecules of cancer-cell-apoptosis: a minireview of progress between Kanglaite injection and related genes. J. Exp. Clin. Cancer Res. 27:31. 10.1186/1756-9966-27-31 18718024 PMC2543009

[B24] Miller K. D. Nogueira L. Mariotto A. B. Rowland J. H. Yabroff K. R. Alfano C. M. (2019). Cancer treatment and survivorship statistic. CA Cancer J. Clin. 69 (5), 363–385. 10.3322/caac.21565 31184787

[B25] Musgrove E. A. Caldon C. E. Barraclough J. Stone A. Sutherland R. L. (2011). Cyclin D as a therapeutic target in cancer. Nat. Rev. Cancer 11 (8), 558–572. 10.1038/nrc3090 21734724

[B26] Nagahashi M. Hait N. C. Maceyka M. Avni D. Takabe K. Milstien S. (2014). Sphingosine-1-phosphate in chronic intestinal inflammation and cancer. Adv. Biol. Regul. 54, 112–120. 10.1016/j.jbior.2013.10.001 24210073 PMC3946530

[B27] Nagahashi M. Yamada A. Katsuta E. Aoyagi T. Huang W. C. Terracina K. P. (2018). Targeting the SphK1/S1P/S1PR1 Axis That Links Obesity, Chronic Inflammation, and Breast Cancer Metastasis. Cancer Res. 78 (7), 1713–1725. 10.1158/0008-5472.CAN-17-1423 29351902 PMC6945803

[B28] Numata M. Yamamoto A. Moribayashi A. Yamada H. (1994). Antitumor components isolated from the Chinese herbal medicine Coix lachryma-jobi. Planta Med. 60 (4), 356–359. 10.1055/s-2006-959500 7938271

[B29] Ogretmen B. Hannun Y. A. (2004). Biologically active sphingolipids in cancer pathogenesis and treatment. Nat. Rev. Cancer 4 (8), 604–616. 10.1038/nrc1411 15286740

[B30] Ogretmen B. (2018). Sphingolipid metabolism in cancer signalling and therapy. Nat. Rev. Cancer 18 (1), 33–50. 10.1038/nrc.2017.96 29147025 PMC5818153

[B31] Paik J. H. Nam S. J. Kim T. M. Heo D. S. Kim C. W. Jeon Y. K. (2014). Overexpression of sphingosine-1-phosphate receptor 1 and phospho-signal transducer and activator of transcription 3 is associated with poor prognosis in rituximab-treated diffuse large B-cell lymphomas. BMC Cancer 14:911. 10.1186/1471-2407-14-911 25472725 PMC4265452

[B32] Purwaha P. Gu F. Piyarathna D. Rajendiran T. Ravindran A. Omilian A. R. (2018). Unbiased Lipidomic Profiling of Triple-Negative Breast Cancer Tissues Reveals the Association of Sphingomyelin Levels with Patient Disease-Free Survival. Metabolites 8 (3), 41. 10.3390/metabo8030041 PMC6161031 30011843

[B33] Pyne S. Pyne N. J. (2011). Translational aspects of sphingosine 1-phosphate biology. Trends Mol. Med. 17 (8), 463–472. 10.1016/j.molmed.2011.03.002 21514226

[B34] Rostami N. Nikkhoo A. Ajjoolabady A. Azizi G. Hojjat-Farsangi M. Ghalamfarsa G. (2019). S1PR1 as a Novel Promising Therapeutic Target in Cancer Therapy. Mol. Diagn. Ther. 23 (4), 467–487. 10.1007/s40291-019-00401-5 31115798

[B35] Rutherford C. Childs S. Ohotski J. McGlynn L. Riddick M. MacFarlane S. (2013). Regulation of cell survival by sphingosine-1-phosphate receptor S1P1 via reciprocal ERK-dependent suppression of Bim and PI-3-kinase/protein kinase C-mediated upregulation of Mcl-1. Cell Death Dis. 4, e927. 10.1038/cddis.2013.455 24263101 PMC3847331

[B36] Schwartzberg L. S. Arena F. P. Bienvenu B. J. Kaplan E. H. Camacho L. H. Campos L. T. (2017). A Randomized, Open-Label, Safety and Exploratory Efficacy Study of Kanglaite Injection (KLTi) plus Gemcitabine versus Gemcitabine in Patients with Advanced Pancreatic Cancer. J. Cancer 8 (10), 1872–1883. 10.7150/jca.15407 28819385 PMC5556651

[B37] Tucci P. Agostini M. Grespi F. Markert E. K. Terrinoni A. Vousden K. H. (2012). Loss of p63 and its microRNA-205 target results in enhanced cell migration and metastasis in prostate cancer. Proc. Natl. Acad. Sci. U. S. A. 109 (38), 15312–15317. 10.1073/pnas.1110977109 22949650 PMC3458363

[B38] Wang L. Kang F. B. Wang J. Yang C. He D. W. (2019). Downregulation of miR-205 contributes to epithelial-mesenchymal transition and invasion in triple-negative breast cancer by targeting HMGB1-RAGE signaling pathway. Anticancer Drugs 30 (3), 225–232. 10.1097/CAD.0000000000000705 30334817 PMC6410973

[B39] Waters C. Sambi B. Kong K. C. Thompson D. Pitson S. M. Pyne S. (2003). Sphingosine 1-phosphate and platelet-derived growth factor (PDGF) act via PDGF beta receptor-sphingosine 1-phosphate receptor complexes in airway smooth muscle cells. J. Biol. Chem. 278 (8), 6282–6290. 10.1074/jbc.M208560200 12480944

[B40] Wei J. Zhang L. Li J. Zhu S. Tai M. Mason C. W. (2017). MicroRNA-205 promotes cell invasion by repressing TCF21 in human ovarian cancer. J. Ovarian Res. 10 (1), 33. 10.1186/s13048-017-0328-1 28476165 PMC5420089

[B41] Weichand B. Popp R. Dziumbla S. Mora J. Strack E. Elwakeel E. (2017). S1PR1 on tumor-associated macrophages promotes lymphangiogenesis and metastasis via NLRP3/IL-1beta. J. Exp. Med. 214 (9), 2695–2713. 10.1084/jem.20160392 28739604 PMC5584110

[B42] Wu Y. Zhang J. Hong Y. Wang X. (2018). Effects of Kanglaite Injection on Serum miRNA-21 in Patients with Advanced Lung Cancer. Med. Sci. Monit. 24, 2901–2906. 10.12659/MSM.909719 29735968 PMC5962241

[B43] Xiao Y. Humphries B. Yang C. Wang Z. (2019). MiR-205 Dysregulations in Breast Cancer: The Complexity and Opportunities. Noncoding RNA 5 (4), 53. 10.3390/ncrna5040053 PMC6958506 31752366

[B44] Yang C. Hou A. Yu C. Dai L. Wang W. Zhang K. (2018). Kanglaite reverses multidrug resistance of HCC by inducing apoptosis and cell cycle arrest via PI3K/AKT pathway. Onco. Targets Ther. 11, 983–996. 10.2147/OTT.S153814 29520149 PMC5833758

[B45] You X. Wang Y. Wu J. Liu Q. Chen D. Tang D. (2018). Galectin-1 Promotes Metastasis in Gastric Cancer Through a Sphingosine-1-Phosphate Receptor 1-Dependent Mechanism. Cell. Physiol. Biochem. Int. J. Exp. Cell. Physiol. Biochem. Pharmacol. 51 (1), 11–30. 10.1159/000495157 30453284

[B46] Zhang H. Fan Q. (2015). MicroRNA-205 inhibits the proliferation and invasion of breast cancer by regulating AMOT expression. Oncol. Rep. 34 (4), 2163–2170. 10.3892/or.2015.4148 26239614

[B47] Zhang X. W. Liu L. Zhang X. Z. Bo P. (2017). Kanglaite inhibits the expression of drug resistance genes through suppressing PVT1 in cisplatin-resistant gastric cancer cells. Exp. Ther. Med. 14 (2), 1789–1794. 10.3892/etm.2017.4650 28810651 PMC5525592

[B48] Zhang P. Meng X. Tang X. Ren L. Liang J. (2019). The effect of a coix seed oil injection on cancer pain relief. Supp. Care Cancer 27 (2), 461–465. 10.1007/s00520-018-4313-z 29971522

