# Supplementary figures and images for: Coix Seed Oil Exerts an Anti–Triple-Negative Breast Cancer Effect by Disrupting miR-205/S1PR1 Axis
Source: Front Pharmacol. 2020 Sep 25;11:529962. doi: 10.3389/fphar.2020.529962 (PMC7556270; doi:10.3389/fphar.2020.529962)

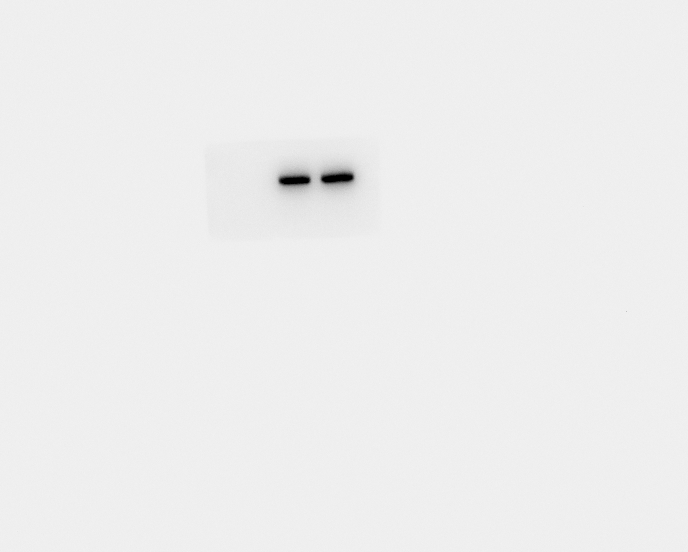

Supplement: Supplementary file 1 [file DataSheet_1.zip › The original image files for the blots-529962/Figure 4-B-GAPDH.tif]

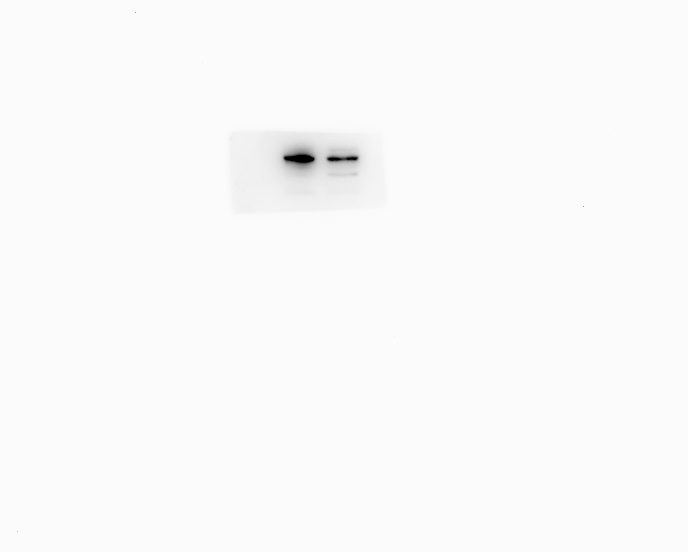

Supplement: Supplementary file 1 [file DataSheet_1.zip › The original image files for the blots-529962/Figure 4-B-S1PR1.tif]

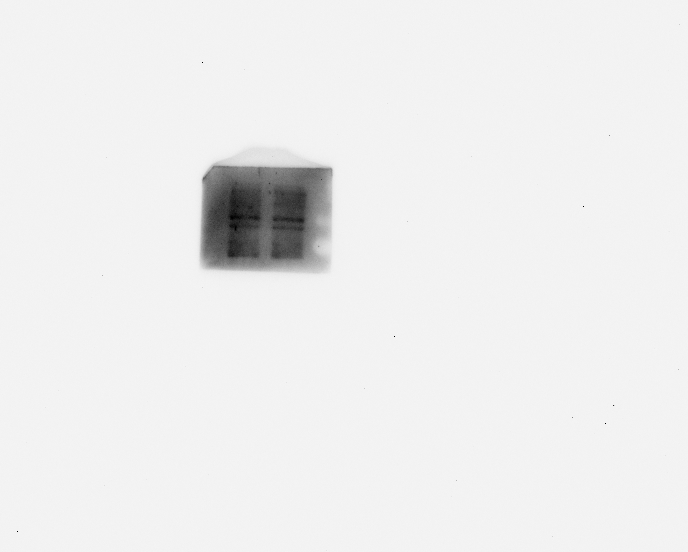

Supplement: Supplementary file 1 [file DataSheet_1.zip › The original image files for the blots-529962/Figure 4-C-p-STAT3.tif]

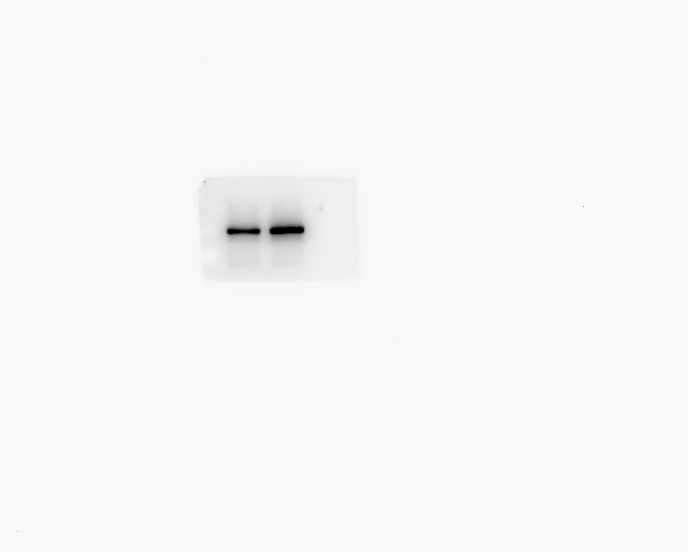

Supplement: Supplementary file 1 [file DataSheet_1.zip › The original image files for the blots-529962/Figure 4-C-STAT3.tif]

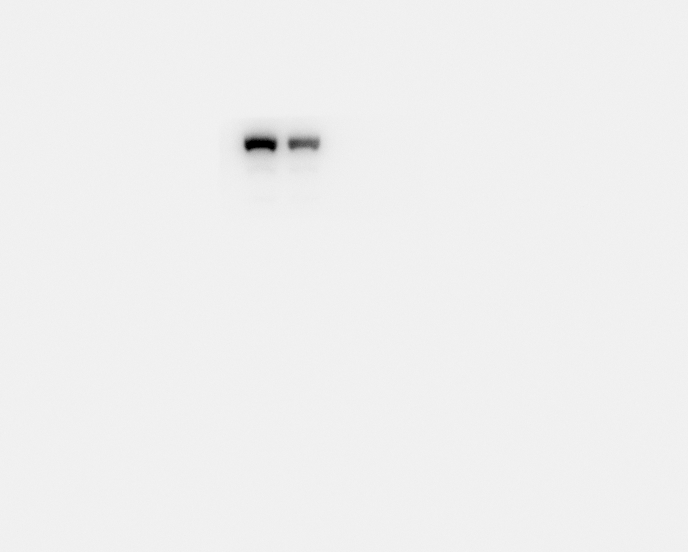

Supplement: Supplementary file 1 [file DataSheet_1.zip › The original image files for the blots-529962/Figure 4-D-Cyclin D1.tif]

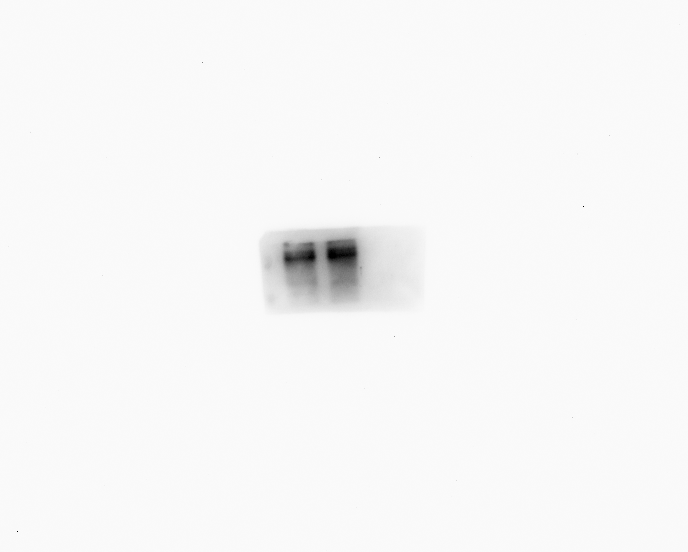

Supplement: Supplementary file 1 [file DataSheet_1.zip › The original image files for the blots-529962/Figure 4-D-p27.tif]

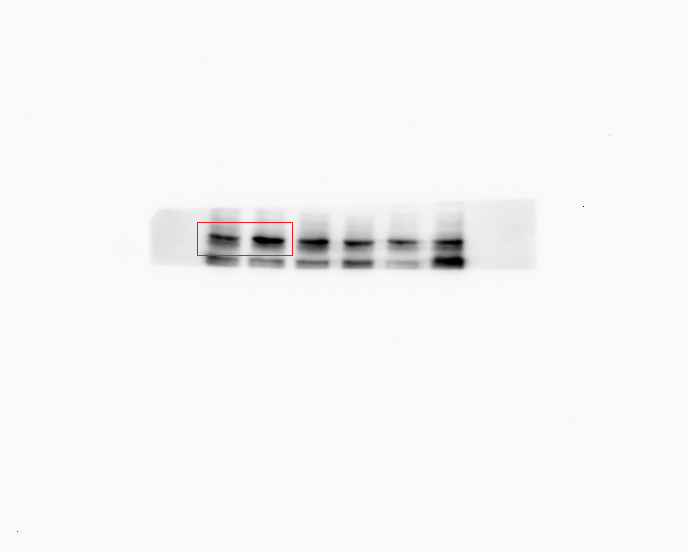

Supplement: Supplementary file 1 [file DataSheet_1.zip › The original image files for the blots-529962/Figure 5-B-GAPDH.tif]

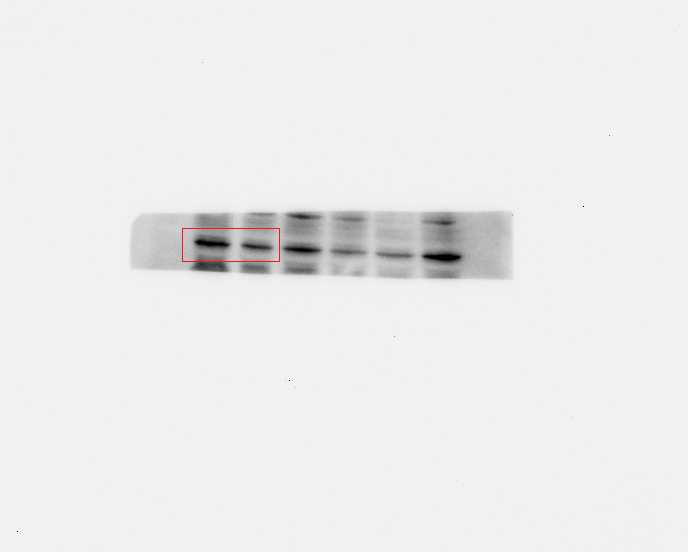

Supplement: Supplementary file 1 [file DataSheet_1.zip › The original image files for the blots-529962/Figure 5-B-S1PR1.tif]

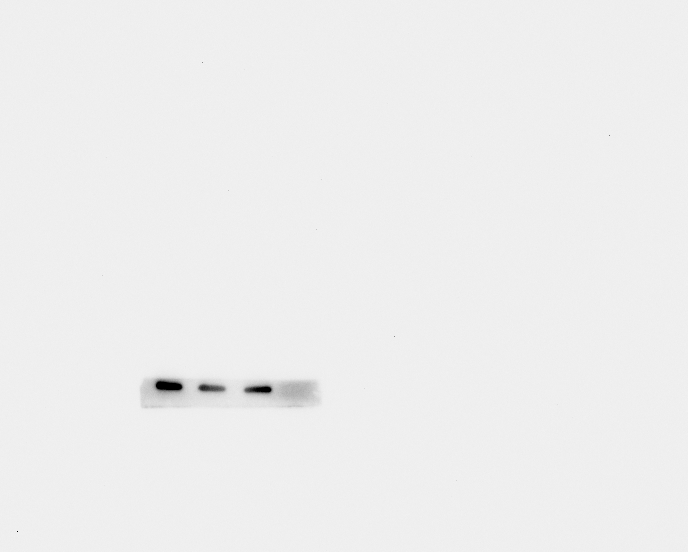

Supplement: Supplementary file 1 [file DataSheet_1.zip › The original image files for the blots-529962/Figure 5-D-GAPDH.tif]

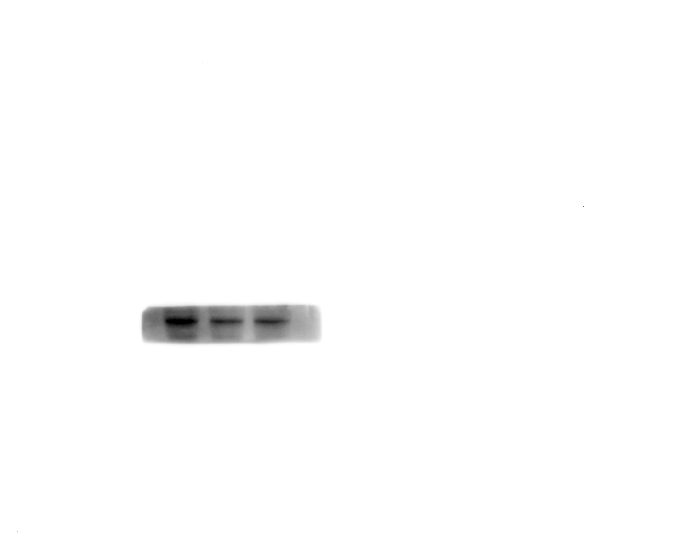

Supplement: Supplementary file 1 [file DataSheet_1.zip › The original image files for the blots-529962/Figure 5-D-S1PR1.tif]

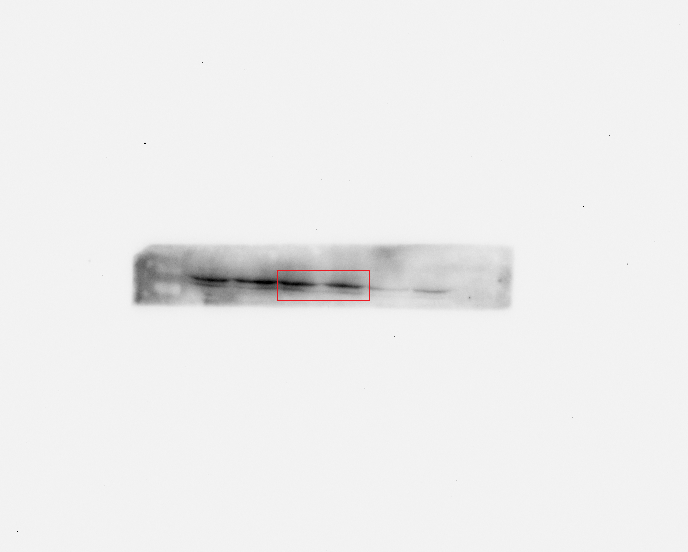

Supplement: Supplementary file 1 [file DataSheet_1.zip › The original image files for the blots-529962/Figure 6-A-p-STAT3.tif]

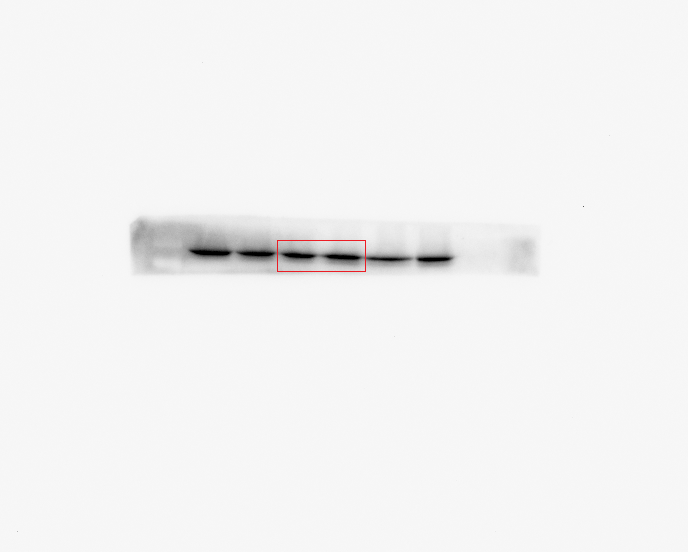

Supplement: Supplementary file 1 [file DataSheet_1.zip › The original image files for the blots-529962/Figure 6-A-STAT3.tif]

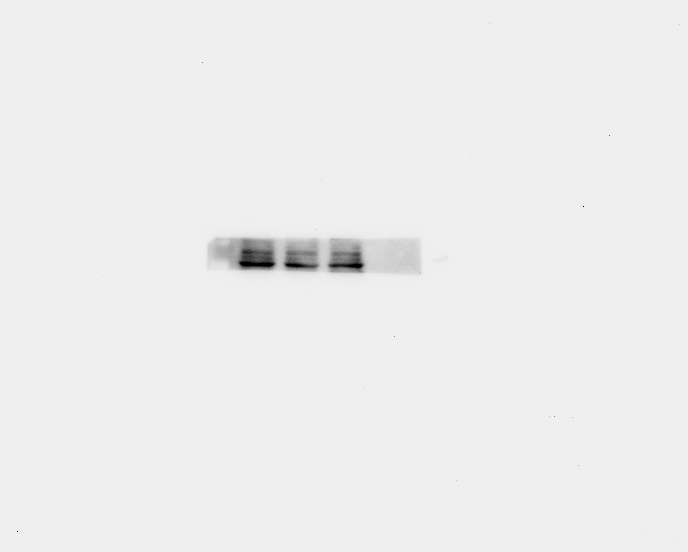

Supplement: Supplementary file 1 [file DataSheet_1.zip › The original image files for the blots-529962/Figure 6-B-p-STAT3.tif]

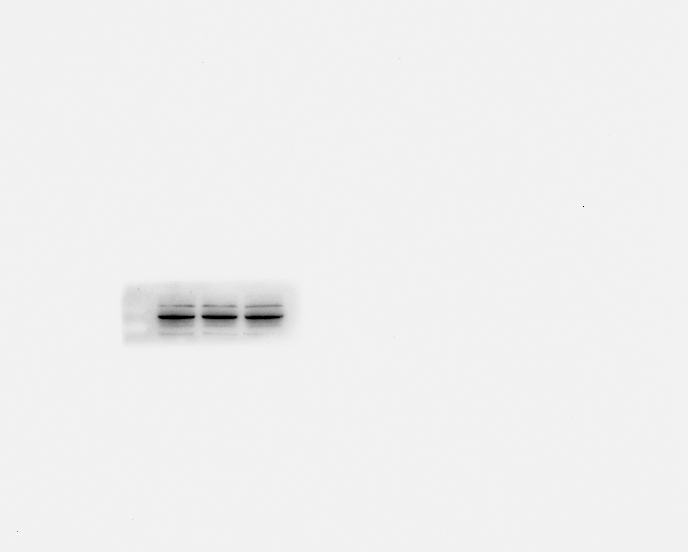

Supplement: Supplementary file 1 [file DataSheet_1.zip › The original image files for the blots-529962/Figure 6-B-STAT3.tif]

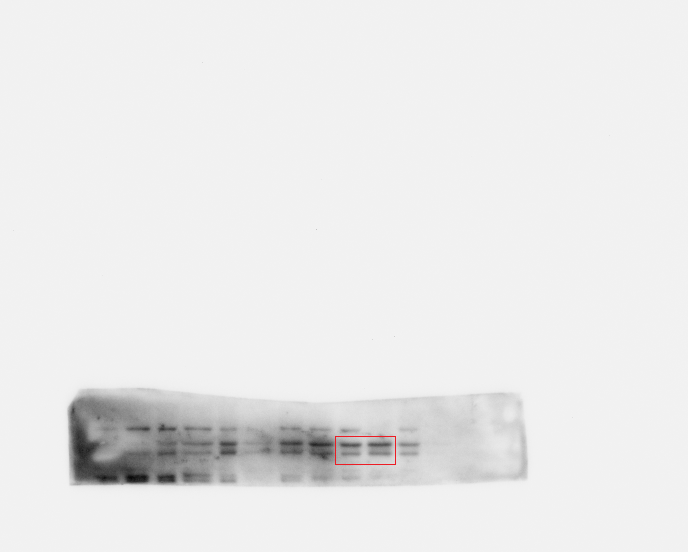

Supplement: Supplementary file 1 [file DataSheet_1.zip › The original image files for the blots-529962/Figure 7-A-ERK.tif]

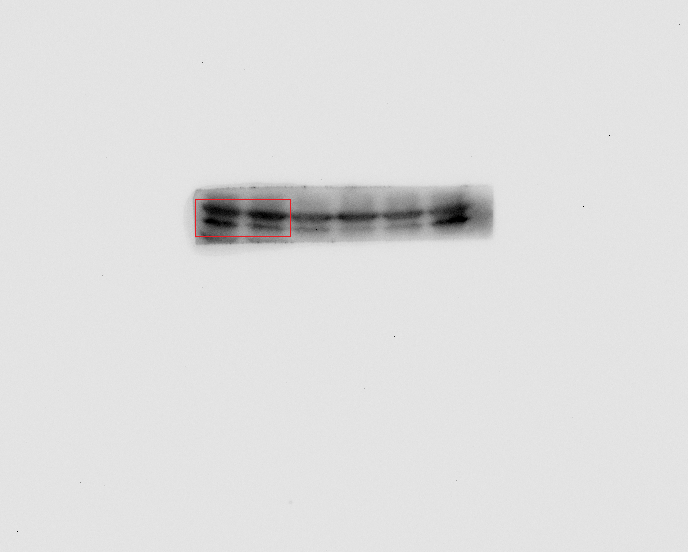

Supplement: Supplementary file 1 [file DataSheet_1.zip › The original image files for the blots-529962/Figure 7-A-JNK.tif]

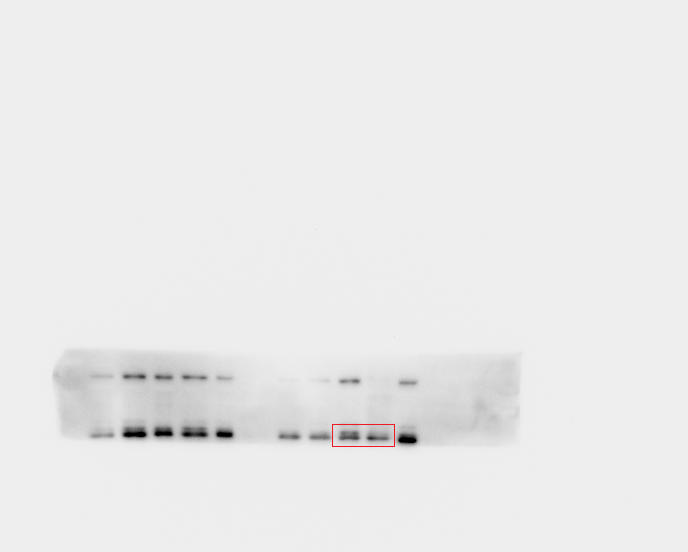

Supplement: Supplementary file 1 [file DataSheet_1.zip › The original image files for the blots-529962/Figure 7-A-p-ERK.tif]

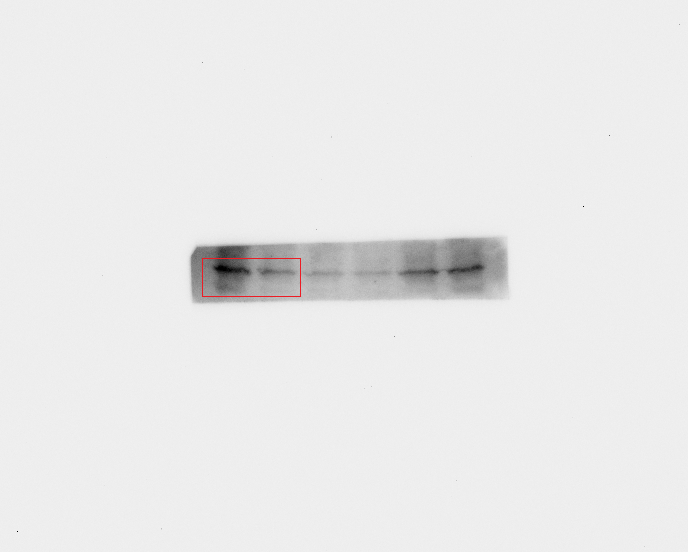

Supplement: Supplementary file 1 [file DataSheet_1.zip › The original image files for the blots-529962/Figure 7-A-p-JNK.tif]

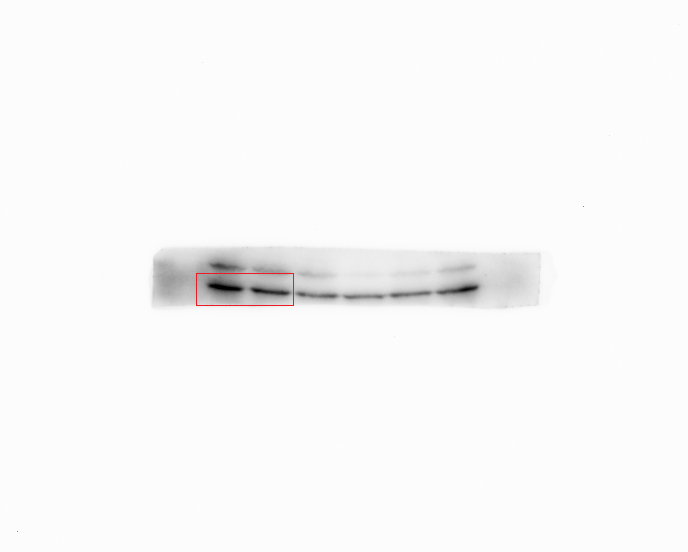

Supplement: Supplementary file 1 [file DataSheet_1.zip › The original image files for the blots-529962/Figure 7-A-p-p38.tif]

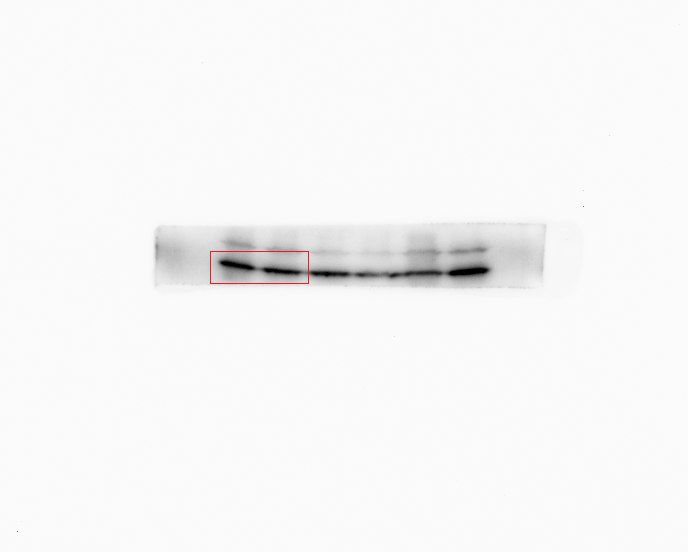

Supplement: Supplementary file 1 [file DataSheet_1.zip › The original image files for the blots-529962/Figure 7-A-p38.tif]

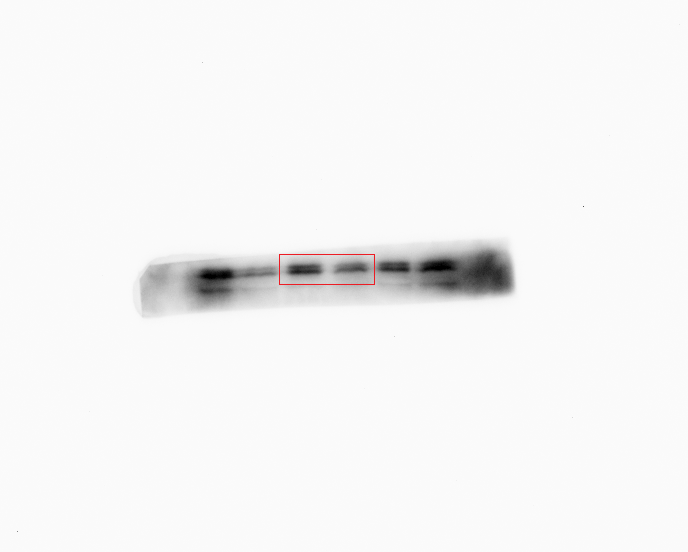

Supplement: Supplementary file 1 [file DataSheet_1.zip › The original image files for the blots-529962/Figure 7-B-Cyclin D1.tif]

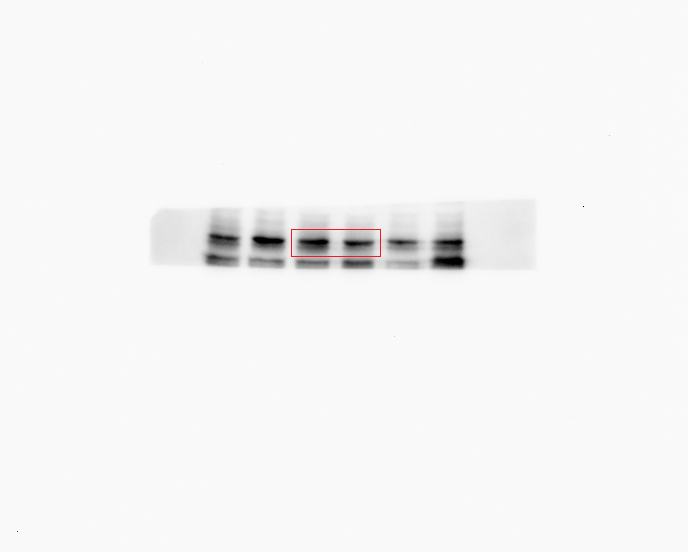

Supplement: Supplementary file 1 [file DataSheet_1.zip › The original image files for the blots-529962/Figure 7-B-GAPDH.tif]

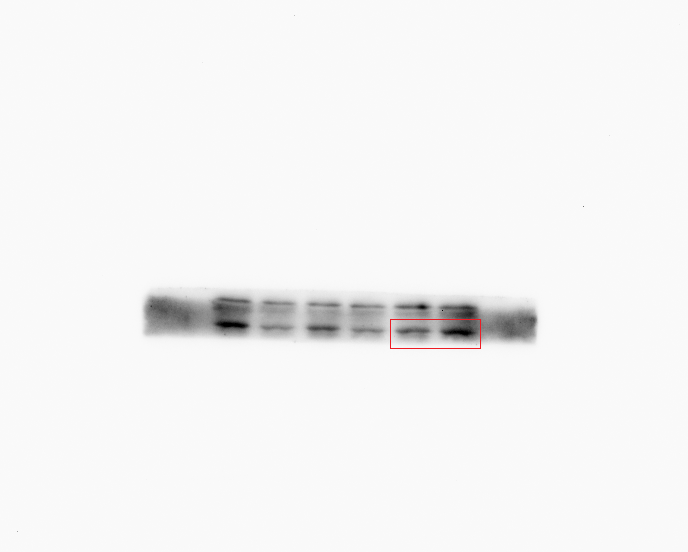

Supplement: Supplementary file 1 [file DataSheet_1.zip › The original image files for the blots-529962/Figure 7-B-p27.tif]

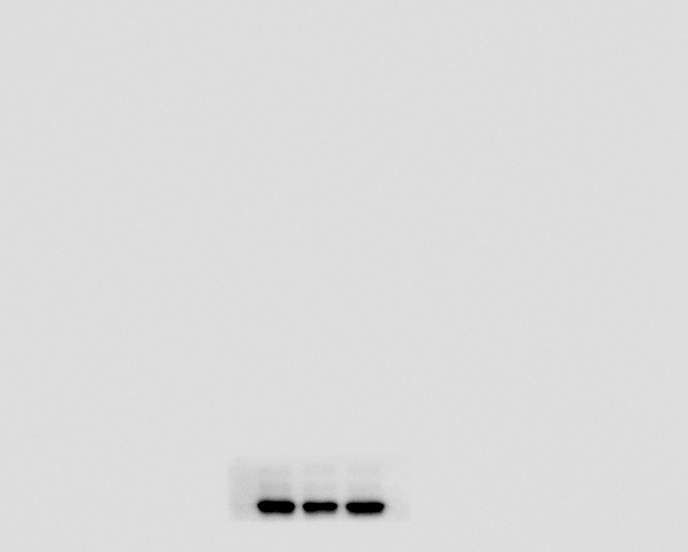

Supplement: Supplementary file 1 [file DataSheet_1.zip › The original image files for the blots-529962/Figure 7-C-AKT.tif]

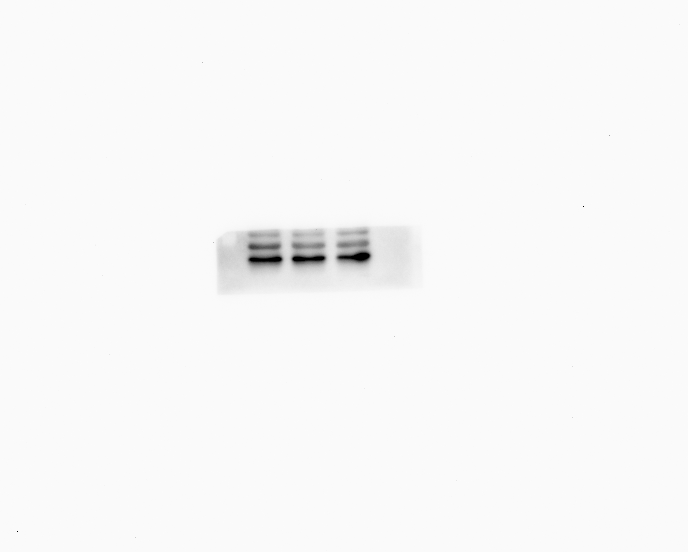

Supplement: Supplementary file 1 [file DataSheet_1.zip › The original image files for the blots-529962/Figure 7-C-ERK.tif]

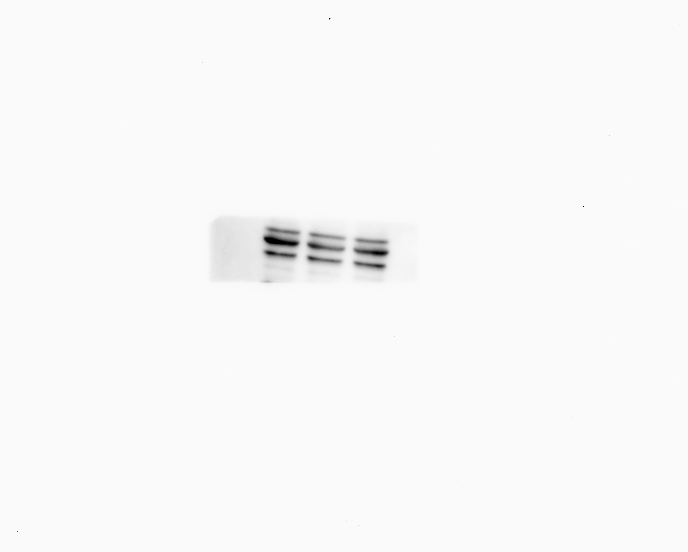

Supplement: Supplementary file 1 [file DataSheet_1.zip › The original image files for the blots-529962/Figure 7-C-JNK.tif]

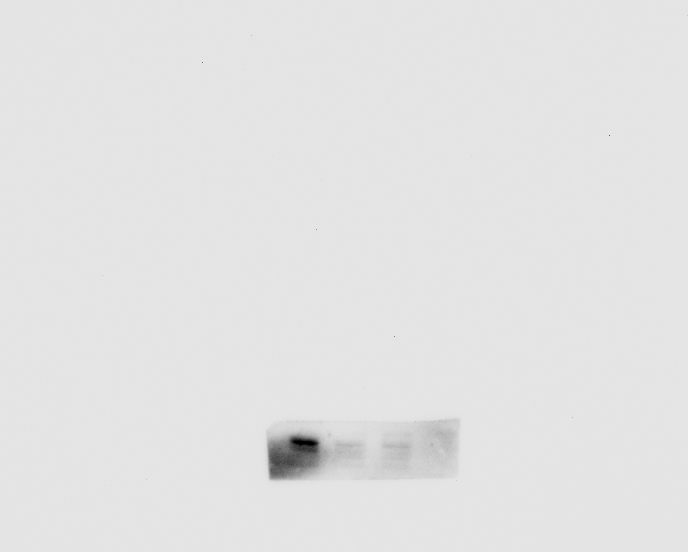

Supplement: Supplementary file 1 [file DataSheet_1.zip › The original image files for the blots-529962/Figure 7-C-p-AKT.tif]

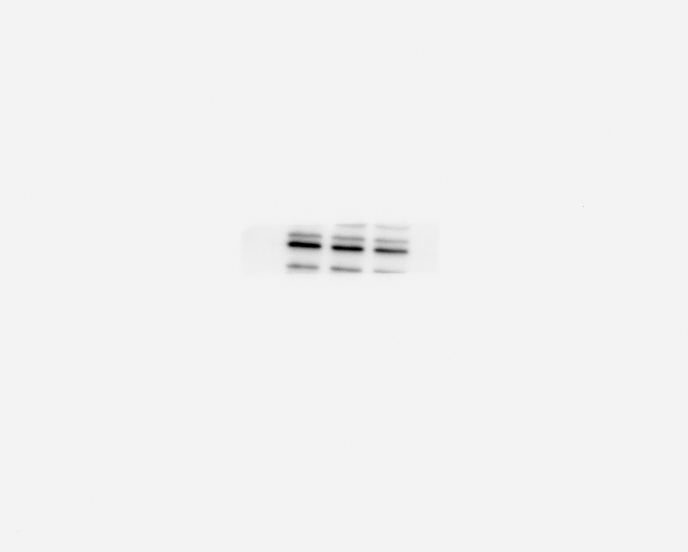

Supplement: Supplementary file 1 [file DataSheet_1.zip › The original image files for the blots-529962/Figure 7-C-p-ERK.tif]

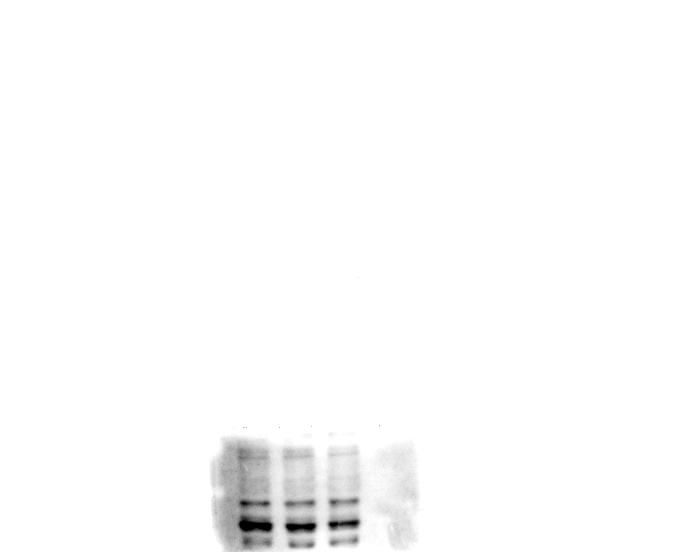

Supplement: Supplementary file 1 [file DataSheet_1.zip › The original image files for the blots-529962/Figure 7-C-p-JNK.tif]

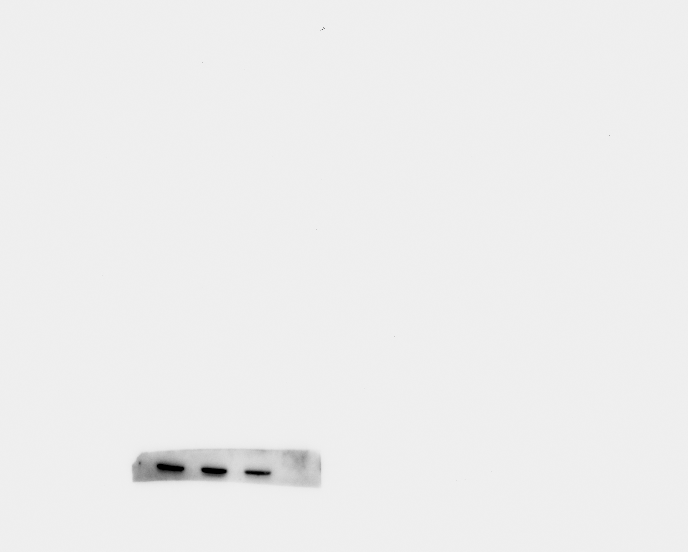

Supplement: Supplementary file 1 [file DataSheet_1.zip › The original image files for the blots-529962/Figure 7-C-p-p38.tif]

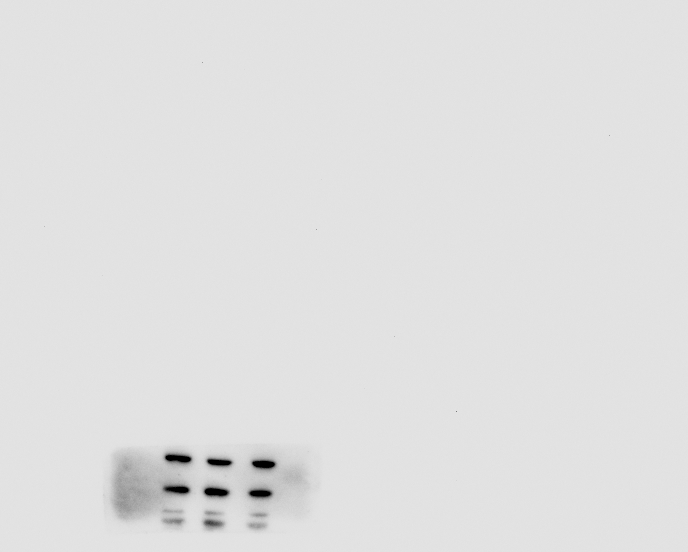

Supplement: Supplementary file 1 [file DataSheet_1.zip › The original image files for the blots-529962/Figure 7-C-p38.tif]

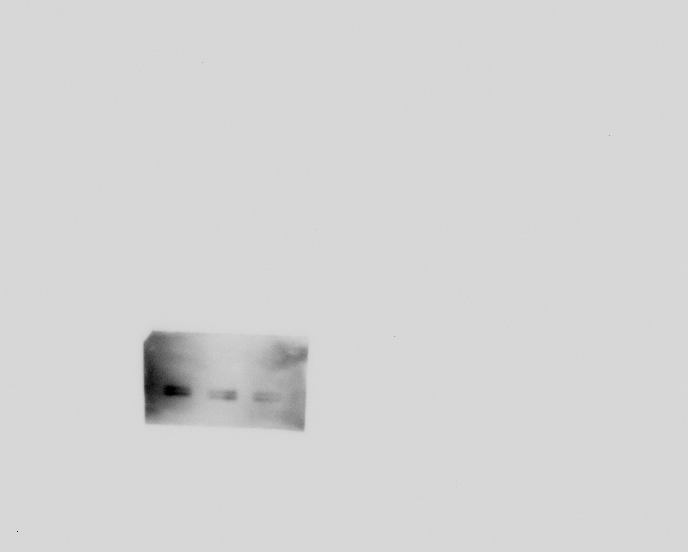

Supplement: Supplementary file 1 [file DataSheet_1.zip › The original image files for the blots-529962/Figure 7-D-Cyclin D1.tif]

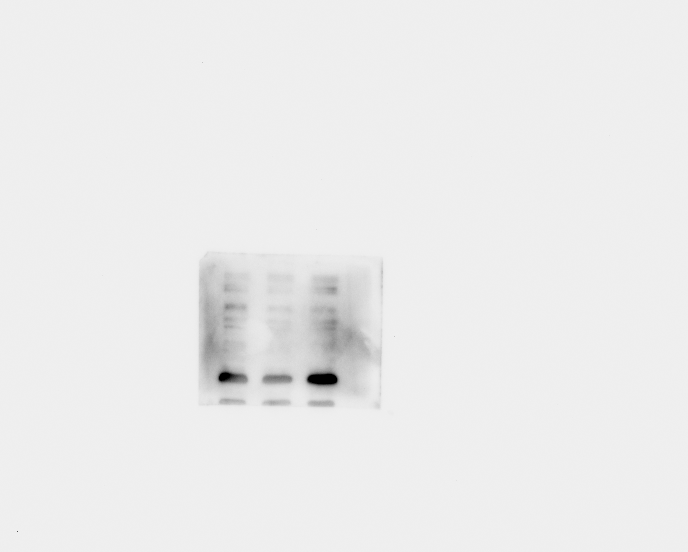

Supplement: Supplementary file 1 [file DataSheet_1.zip › The original image files for the blots-529962/Figure 7-D-p27.tif]
