# Supplementary material for: Coix Seed Oil Exerts an Anti–Triple-Negative Breast Cancer Effect by Disrupting miR-205/S1PR1 Axis
Source: Front Pharmacol. 2020 Sep 25;11:529962. doi: 10.3389/fphar.2020.529962 (PMC7556270; doi:10.3389/fphar.2020.529962)
Supplement: Supplementary file 2 [file Table_1.docx]

**Supplementary Materials**

**The identification of six compounds**

LC-MS was used to test the Coix Seed oil used in the experiment. The results showed that the sample had a corresponding effect in the positive ion mode, and six compounds were detected in ESI^+^ mode. Their retention times and pseudo molecular ion peak of the observation were 17.09 min, *M/z* 872.77 [M+NH4^+^]^+^ (1), 17.27 min, *M/z* 898.79 [M+NH4^+^]^+^ (2), 17.71 min, *M/z* 874.79 [M+NH4^+^]^+^ (3), 17.85 min, *M/z* 900.80 [M+NH4^+^]^+^ (4), 18.26 min, *M/z* 876.80 [M+NH4^+^]^+^ (5), 18.40 min, M/z 902.82 [M+NH4^+^]^+^ (6). Six compounds were identified by comparing retention time and MS data with compounds contained in Coix Seed oil, they were 1, 2-Dilinoleoyl-3-palmitoyl-rac-glycerol, 1, 2- Dilinoleoyl-3-oleoyl-rac-glycerol, 1-palmitoyl-2-oleoyl-3-linoleoyl-rac-glycerol, 1, 2-Dioleoyl-3-linoleoyl-rac-glycerol, 1, 2-Dioleoyl-3-palmitoyl-rac-glycerol and Glycerol trioleate.


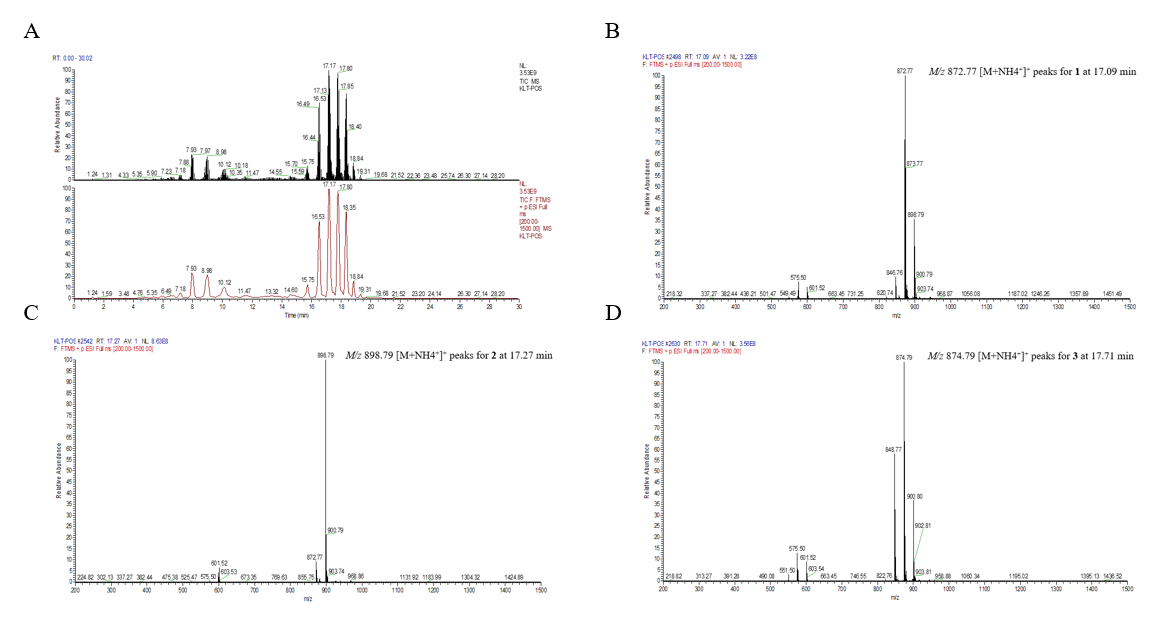


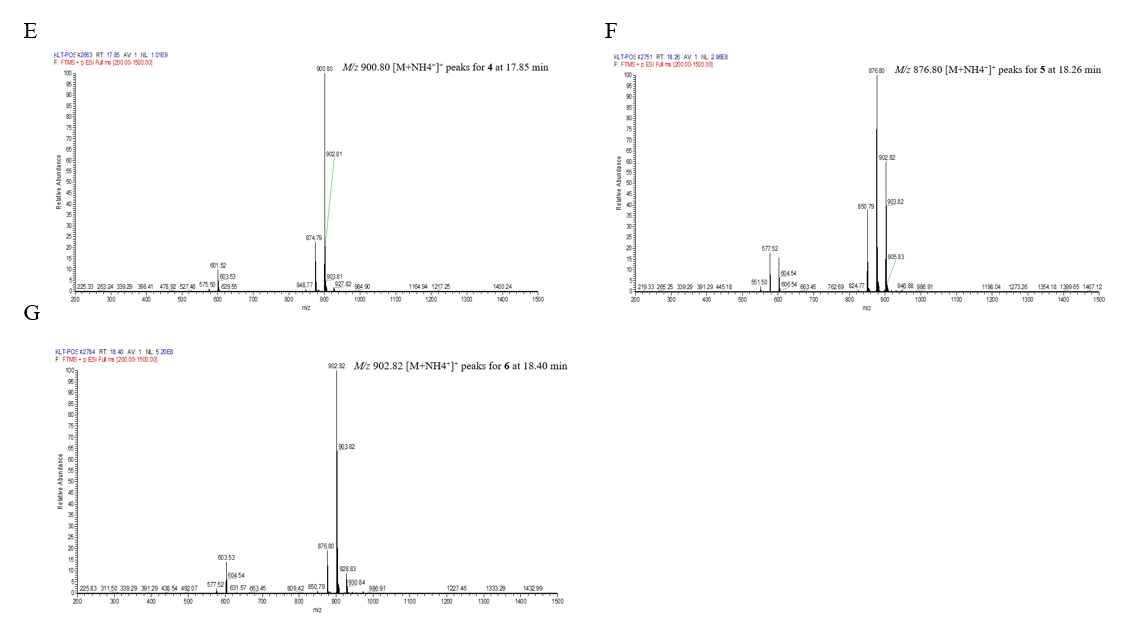


**Figure S1.** LC-MS of Coix Seed oil in positive mode (A). (B) ESI–MS spectra of [M+NH4^+^]^+^ ion of compound 1 (retention time: 17.09 min). (C) ESI–MS spectra of [M+NH4^+^]^+^ ion of compound 2 (retention time: 17.27 min). (D) ESI–MS spectra of [M+NH4^+^]^+^ ion of compound 3 (retention time: 17.71 min). (E) ESI–MS spectra of [M+NH4^+^]^+^ ion of compound 4 (retention time: 17.85 min). (F) ESI–MS spectra of [M+NH4^+^]^+^ ion of compound 5 (retention time: 18.26 min). (G) ESI–MS spectra of [M+NH4^+^]^+^ ion of compound 6 (retention time: 18.40 min).

**Effects of ten kinds of long-chain fatty acids with high content in Coix Seed oil on cell viability**

As shown in the previous results, by LC-MS results of Coix Seed oil, we found several higher levels of long-chain fatty acids. In order to further investigate the anticancer potential of these triglycerides, we tested the effects of ten triglycerides on the viability of triple-negative mammary carcinoma cells *in vitro* at different concentrations. As shown in **Figure S2**, only three drugs had a good inhibitory effect on 4T1 cells, while the other seven were basically ineffective. Three of them are Oleic acid, Linoleic acid, Palmitic acid and the other seven are Glycerol trioleate, 1-palmitoyl-2-oleoyl-3-linoleoyl-rac-glycerol, 1,2-Dilinoleoyl-3-oleoyl-rac-glycerol, 1,2-Dilinoleoyl-3-palmitoyl-rac-glycerol, 1,2-Dioleoyl-3-linoleoyl-rac-glycerol, 1,2-Dioleoyl-3-palmitoyl-rac-glycerol and Stearic acid.


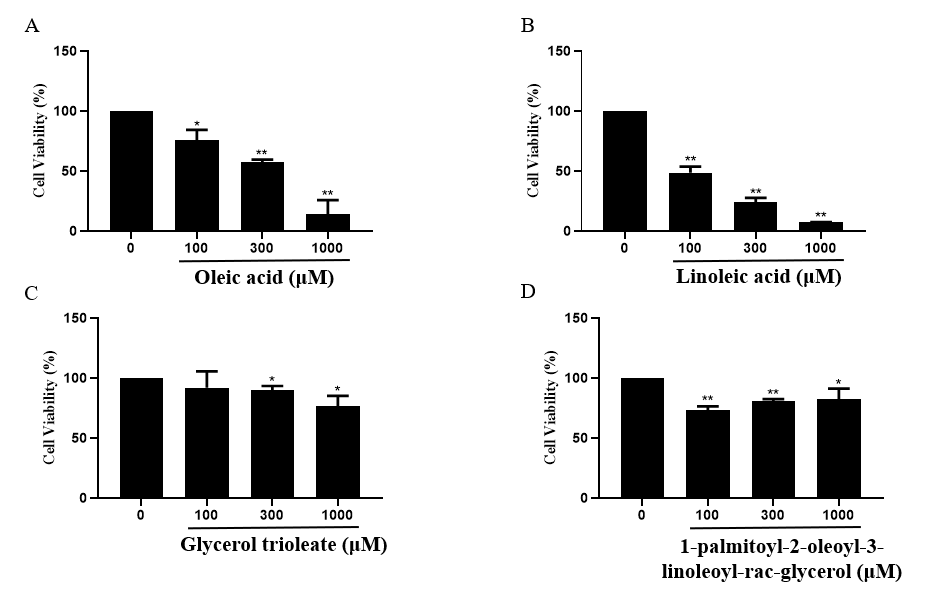

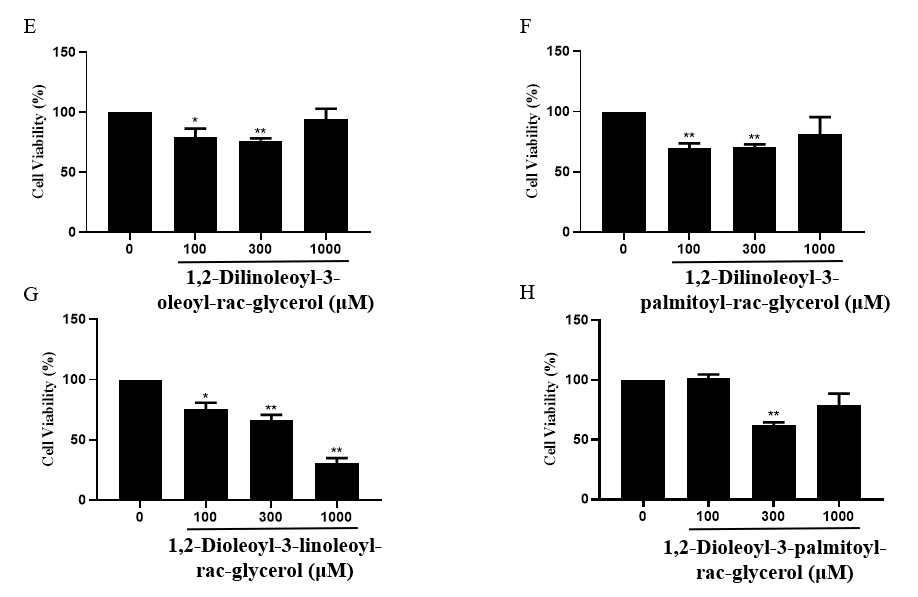

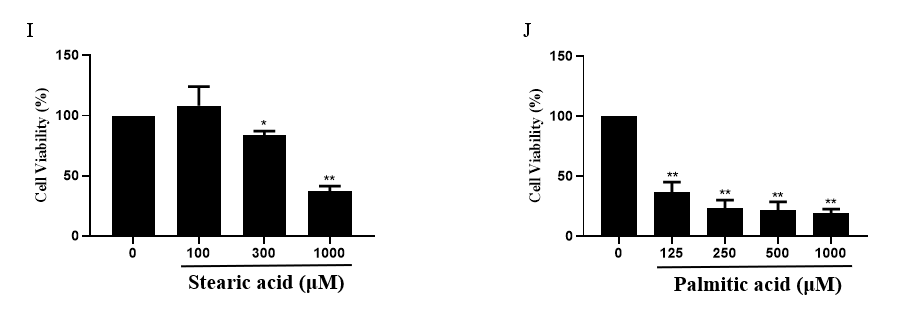


**Figure S2.** Effect of triglycerides on cell viability *in vitro*. (A) Effects of Oleic acid on 4T1 cell viability. (B) Effects of Linoleic acid on 4T1 cell viability. (C) Effects of Glycerol trioleate on 4T1 cell viability. (D) Effects of 1-palmitoyl-2-oleoyl-3-linoleoyl-rac-glycerol on 4T1 cell viability. (E) Effects of 1,2-Dilinoleoyl-3-oleoyl-rac-glycerol on 4T1 cell viability. (F) Effects of 1,2-Dilinoleoyl-3-palmitoyl-rac-glycerol on 4T1 cell viability. (G) Effects of 1,2-Dioleoyl-3-linoleoyl-rac-glycerol on 4T1 cell viability. (H) Effects of 1,2-Dioleoyl-3-palmitoyl-rac-glycerol on 4T1 cell viability. (I) Effects of Stearic acid on 4T1 cell viability. (J) Effects of Palmitic acid on 4T1 cell viability. **p<*0.05, ***p*<0.01 when compared to the untreated.

**Effects of Coix Seed oil on tumor growth of MDA-MB-231 breast cancer**

In order to evaluate the effect of Coix Seed oil on the growth of another triple-negative breast cancer tumor, a nude mouse orthotopic allograft transplantation model based on triple-negative MDA-MB-231 cell was used. As shown in **Figure S3**, compared with the untreated group, Coix Seed oil significantly inhibited the growth of MDA-MB-231 tumor *in vivo* (*p*<0.05). There was no significant difference in body weight of mice between the control and treatment groups. These results indicated that Coix Seed oil has an inhibitory effect on the growth of triple negative breast cancer in another tumor model.


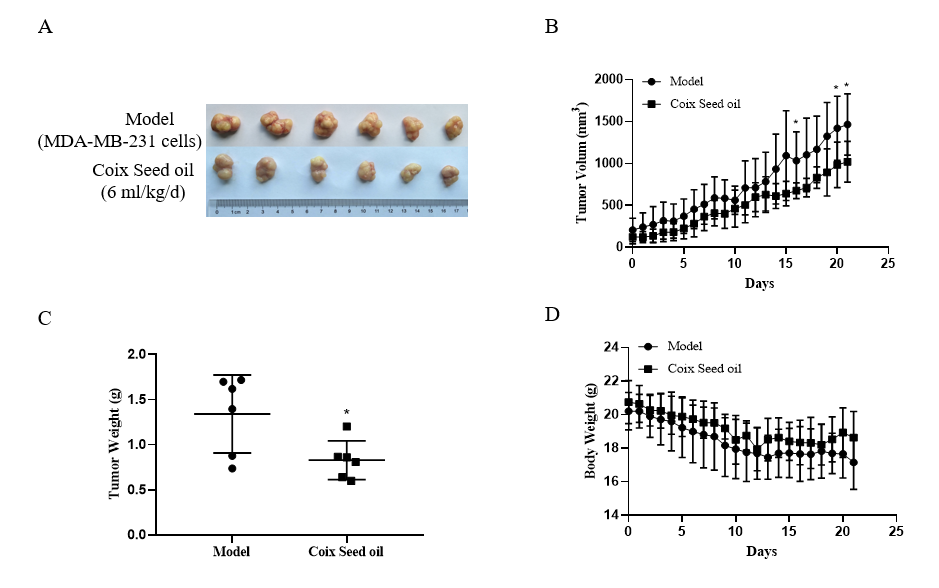


**Figure S3.** Effect of Coix Seed oil on tumor growth of MDA-MB-231 breast cancer. (A) Tumor image. Nude mice were inoculated with MDA-MB-231 cells and treated with Coix Seed oil for 21 days. The tumor was excised and image taken separately. (B) tumor volume for the MDA-MB-231 cell model. (C) Tumor weight for the MDA-MB-231 cell model. (D) Body weight for the MDA-MB-231 cell model. **p<*0.05 when compared to the untreated.
